# Environmental Exposures and Mammary Gland Development: State of the Science, Public Health Implications, and Research Recommendations

**DOI:** 10.1289/ehp.1002864

**Published:** 2011-06-22

**Authors:** Ruthann A. Rudel, Suzanne E. Fenton, Janet M. Ackerman, Susan Y. Euling, Susan L. Makris

**Affiliations:** 1 Silent Spring Institute, Newton, Massachusetts, USA; 2 National Toxicology Program, National Institute of Environment Health Sciences, National Institutes of Health, Department of Health and Human Services, Research Triangle Park, North Carolina, USA; 3 National Center for Environmental Assessment, U.S. Environmental Protection Agency, Washington, DC, USA

**Keywords:** breast cancer, carcinogen susceptibility, development, endocrine disruptors, human health, lactation, mammary gland, risk assessment, rodent model, whole mount

## Abstract

Objectives: Perturbations in mammary gland (MG) development may increase risk for later adverse effects, including lactation impairment, gynecomastia (in males), and breast cancer. Animal studies indicate that exposure to hormonally active agents leads to this type of developmental effect and related later life susceptibilities. In this review we describe current science, public health issues, and research recommendations for evaluating MG development.

Data sources: The Mammary Gland Evaluation and Risk Assessment Workshop was convened in Oakland, California, USA, 16–17 November 2009, to integrate the expertise and perspectives of scientists, risk assessors, and public health advocates. Interviews were conducted with 18 experts, and seven laboratories conducted an MG slide evaluation exercise. Workshop participants discussed effects of gestational and early life exposures to hormonally active agents on MG development, the relationship of these developmental effects to lactation and cancer, the relative sensitivity of MG and other developmental end points, the relevance of animal models to humans, and methods for evaluating MG effects.

Synthesis: Normal MG development and MG carcinogenesis demonstrate temporal, morphological, and mechanistic similarities among test animal species and humans. Diverse chemicals, including many not considered primarily estrogenic, alter MG development in rodents. Inconsistent reporting methods hinder comparison across studies, and relationships between altered development and effects on lactation or carcinogenesis are still being defined. In some studies, altered MG development is the most sensitive endocrine end point.

Conclusions: Early life environmental exposures can alter MG development, disrupt lactation, and increase susceptibility to breast cancer. Assessment of MG development should be incorporated in chemical test guidelines and risk assessment.

The trend toward earlier breast development initiation in U.S. girls (Euling et al. 2008) may put them at increased risk of later life outcomes such as breast cancer—already the most common cancer in U.S. women (American Cancer Society 2010) and a leading cause of death for U.S. women in midlife (Brody et al. 2007). Although many factors, such as nutritional status and body size, may contribute to maturation trends (Kaplowitz 2008) and breast cancer (Renehan et al. 2008), environmental chemicals have been hypothesized to contribute as well (Birnbaum and Fenton 2003; Brody et al. 2007; Euling et al. 2008). Animal studies demonstrate that early life exposure to hormonally active agents can lead to effects on mammary gland (MG) development, impaired lactation, and increased susceptibility to cancer (Fenton 2006). However, the influence of environmental exposures on breast development outcomes is poorly understood, as is the relationship between breast development, lactational deficits, and breast cancer. Few chemicals coming into the marketplace are evaluated for these effects. The findings in animal studies raise concerns that perturbations to human breast development may increase the risk for later life adverse effects including lactation impairment, gynecomastia (in males), and breast cancer in either sex.

This review is the result of the Mammary Gland Evaluation and Risk Assessment Workshop held in Oakland, California, USA, on 16–17 November 2009 to review and discuss recent findings of altered MG development after gestational/perinatal exposure to certain endocrine-disrupting chemicals (EDCs). Workshop participants included research scientists from multiple disciplines, public health advocates, and risk assessors. Many of the participants are leading internationally recognized MG experts.

The goal of the workshop was to improve assessment of MG developmental end points and their integration into human health risk assessment. Workshop participants discussed current research on the effects of developmental exposures to EDCs on MG development, the relationship of these effects to later life lactation and cancer outcomes, relative sensitivity of MG development and other developmental reproductive end points, relevance of effects in animal models to humans, and MG assessment in current toxicology protocols. Data gaps and research recommendations were identified at the workshop and through interviews with 18 risk assessors and toxicologists. Presentation slides and other workshop materials are available online (Silent Spring Institute 2011). In conjunction with the workshop, experts from seven laboratories in Canada, the United States, and Argentina participated in a round-robin evaluation of MG whole mounts.

## Factors Affecting Mammary Gland Development

**Figure 1:**
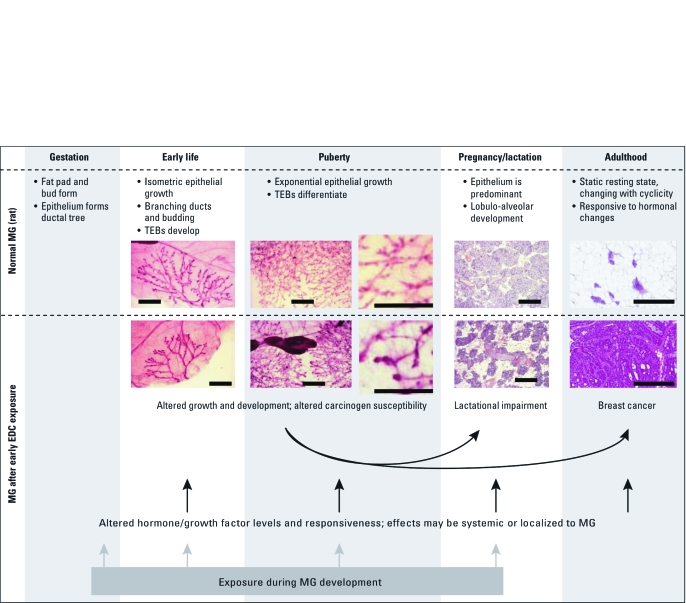
Stages of normal rat MG development and effects of environment on subsequent events. Effects of early life EDC exposures can lead to altered developmental programming in the breast and have been reported neonatally, at puberty, and well into adulthood, when effects on lactation or mammary tumorigenesis become evident. The normal morphology and pace of pubertal development are often altered, and these effects can be observed using MG whole-mount preparations. Transient or permanent effects may be due to gene imprinting, altered gene expression, modified endogenous MG signaling, or changes in hormonal milieu. Arrows indicate plausible (black) or more certain (gray) mechanistic pathways. Photomicrographs for early life and puberty were all taken at 16× magnification on a macroscope (adapted from Enoch et al. 2007, with permission from *Environmental Health Perspectives*); photomicrographs for pregnancy/lactation and adulthood were taken at 10× magnification on a standard microscope (from S.E.F.). Bars = 2 mm.

The female MG undergoes most of its development postnatally, achieving a fully differentiated state late in pregnancy. This process includes numerous events that can be disrupted by exposure to EDCs. Gestation, puberty, and pregnancy are the critical periods during which EDC exposure may most affect MG development (Fenton 2006). Critical events include mammary bud development in the fetus, exponential epithelial outgrowth during puberty, and the rapid transition to lactational competency that occurs during late pregnancy (Figure 1). These stages occur in both rodents and humans.

*Normal MG development.* Normal female MG development involves a well-orchestrated sequence of events marked by extensive proliferation at puberty and by proliferation and differentiation during pregnancy. This process is regulated by hormones, growth factors, and stromal factors and is similar between rodents and humans, although rodent MG development is more completely described (Kleinberg et al. 2009; Medina 2005). Female human MG development begins with budding and branching between 6 and 20 weeks of gestation, yielding at birth a primitive gland composed of ducts ending in ductules. During childhood, MG growth keeps pace with overall body growth; at puberty it accelerates dramatically.

In rodents, the epithelial bud is formed at the site of the nipple around gestation days (GDs) 12–16, and by birth the epithelium has entered the fat pad and formed a ductal tree. The fat pad and mammary epithelium grow at the same pace as the body for the first 2–3 weeks of life, and just before puberty, an exponential growth phase begins. In rodents, and presumably in humans, this phase of ductal development is characterized by formation of terminal end buds (TEBs), which lead the epithelial extension through the fat pad, leaving behind a network of branched ducts. After the fat pad is filled, TEBs differentiate into terminal ductal structures, namely, terminal ductal lobular units in humans, lobules and alveolar buds in rats, and terminal ducts in mice. In humans and rodents, additional MG proliferation and regression events occur with each luteal phase of the ovulatory cycle, and at pregnancy there is significant differentiation of the terminal structures with lobular–alveolar development (Kleinberg et al. 2009; Russo and Russo 2004a, 2004b). Mammary epithelial growth also occurs in male rats and men, whereas male mice lack mammary epithelium. Male mice and rats do not normally possess nipples because androgens during gestation induce regression. Retained nipples in male rats is a characteristic effect of prenatal antiandrogen exposure (Foley et al. 2001).

*Assessment of altered MG development.* Whole mounts and other techniques. Early life treatment with some hormonally active agents results in altered development of the MG in male and female rodents. Although laboratories vary in their methods for reporting altered MG development, the primary approach has been morphological assessment of the entire fourth or fifth abdominal MG fat pad mounted flat on a slide, fixed, stained, defatted, and permanently affixed to the slide as a “whole mount.” Whole mounts allow an assessment of total and relative abundance of mammary terminal ductal structures (i.e., TEBs, terminal ducts, alveolar buds, and lobules), extension of the epithelial cells through the fat pad, and branching patterns and density at different times during development [e.g., Fenton et al. 2002; see also Supplemental Material, Table 1 (doi:10.1289/ehp.1002864)]. A common measurement in mammary whole mounts is the number of TEBs. A TEB is a teardrop-shaped duct end with a diameter of about 100 µm in the rat compared with about 70 µm for a terminal duct (Russo and Russo 1978).

**Table 1:** Female MG outcomes after developmental environmental exposures: rodent–human concordance for selected agents.

Human study MG outcomes					Animal study MG outcomes				
Environmental factor	Development	Lactation	Cancer risk	Development	Lactation	Cancer susceptibility
Hormonal milieu: dosing (animals) or surrogates (humans)		Δ		Δ		Δ		Δ		Δ (EE_2_-dams),*a *— (EE_2_-offspring)*b *		Δ
DES						Δ*c *		Δ		Δ (Dams)		Δ
Genistein/soy		Δ				Δ		Δ		Δ (Dams)		
										Δ (Offspring)		Δ
DDT/DDE				Δ*c *		Δ		Δ		– (Dams)		
Dioxins/furans		Δ				Δ*d *		Δ		Δ (Dams)		Δ
Abbreviations: —, no effect on this end point; Δ, at least one study has reported an association between the exposure and altered outcomes [see details and citations in Supplemental Material, Table 2 (doi:10.1289/ehp.1002864)]; DDE, dichlorodiphenyldichloroethylene; DDT, dichlorodiphenyltrichloroethane; DMBA, dimethylbenzanethrene; EE_2_, ethinylestradiol. Examples of concordance between rodents and humans for MG effects are included here. In some cases, findings are mixed or conflicting; in human studies, exposure measures are often imprecise. **a**Lactation effect in animals dosed continuously or during pregnancy and/or lactation. **b**Lactation effect in animals dosed only *in utero* and/or preweaning. **c**Conflicting findings. **d**Exposure may not have been during early life/development.												

Several rodent studies have reported altered MG development after prenatal, neonatal, or peripubertal exposure to a range of hormonally active agents, including pharmaceutical hormones, dietary constituents, and EDCs [see Supplemental Material, Table 1 (doi:10.1289/ehp.1002864)]. These studies typically include histopathological evaluation of MG whole mounts of developing animals and report morphological features such as branching, extent of growth, and relative proportion of structures (e.g., TEBs, lobules, and terminal ducts). Other studies report changes in morphology or immunohistochemistry of tissue sections or gene expression in tissue homogenates. Methods and data reporting vary between laboratories, making it difficult to compare findings across studies. More uniform approaches will facilitate progress; however, unanticipated end points should continue to be reported because this field is still developing.

Morphological changes reflect timing  of assessment. Because normal development involves a well-characterized, consistent progression of types and ratios of terminal structures and extension through the fat pad, alterations are sometimes reported as accelerated or delayed development relative to controls (e.g., Moon et al. 2007). Some agents alter the pace at which differentiation occurs, leading to an increased or decreased number of TEBs depending on timing of assessment. If a perinatally administered EDC causes accelerated development, the number of TEBs in the treated group will be higher than that in vehicle-treated controls at weaning [postnatal day (PND) 21] because of increased proliferation, but lower in early adulthood (PNDs 45–50) because of accelerated differentiation, as is seen after exposure to estrogens (Hovey et al. 2005). In the case of an EDC, such as dioxin, that causes delayed development, reduced differentiation leads to a higher number of TEBs in early adulthood and a longer period during which TEBs are present (Brown et al. 1998; Fenton et al. 2002). The number of TEBs present in the gland also depends on the number of ducts in the gland. Therefore, the number of TEBs at a particular time point can be altered by changes to the extent of growth as well as to the pace of differentiation. For example, if the overall number of ducts is decreased by an environmental exposure, then the overall number of TEBs in the gland will be decreased compared with those in controls at any time point, as demonstrated for perfluorooctanoic acid (PFOA) exposures in mice (White et al. 2009). Some reports have not differentiated between changes in TEB number due to overall gland size and those due to altered developmental pace. Evaluation at multiple time points and consideration of the total number of terminal ends, as well as the absolute number of TEBs, alleviate this problem and convey the relative number of structures.

*Steroid hormones.* In one of the first studies  of neonatal exposure to estrogen, progesterone, or both in mice, Jones and Bern (1979) reported irreversible adult MG effects, including secretory stimulation, dilated ducts, and abnormal lobuloalveolar development. Perinatal treatment with estrogens such as estradiol or diethylstilbestrol (DES) has been reported to produce accelerated development, characterized by increased pubertal TEB density, and to promote ductal proliferation during the peripubertal period in both rats and mice (Fielden et al. 2002; Hilakivi-Clarke et al. 1997; Hovey et al. 2005; Tomooka and Bern 1982; Warner 1976). In addition, Doherty et al. (2010) reported that prenatal DES exposure in mice altered expression in MG of genes that may be important in tumorigenesis. Ovariectomy has been reported to diminish or obviate the effect of neonatal ovarian steroids on mouse MG development (Jones and Bern 1979; Mori et al. 1976), and strain differences in sensitivity have also been reported (Mori et al. 1976; Yang et al. 2009). In rats exposed continuously beginning at conception, oral ethinyl estradiol exposure induced ductal hyperplasia in male rat MGs by PND50, and this effect was less apparent in rats assessed later in life (Latendresse et al. 2009). Thus, morphological changes in MG reflect timing of exposure as well as timing of assessment, and so both of these variables must be considered when comparing results across studies. Supplemental Material, Table 1 (doi:10.1289/ehp.1002864) compiles the methods and findings of studies that have evaluated the effects of hormone, dietary, or chemical exposures during the prenatal, neonatal, or peripubertal periods on MG development up to 10 weeks. Several additional endocrine-sensitive end points commonly assessed to indicate relative sensitivity are also included in the table.

In addition, whereas perinatal steroid hormone exposure alters proliferation and TEB number, peripubertal exposure that occurs after proliferation has begun affects mainly the differentiation of TEBs into mature structures. For example, pubertal DES treatment in rats increased the pace of lobule formation and decreased the number of terminal ducts and TEBs compared with vehicle-treated controls just after puberty (Odum et al. 1999). Prepubertal DES treatment of rats (on PNDs 23–29) resulted in fewer TEBs, terminal ducts, and alveolar buds, with a concomitant increase in the more differentiated lobules, overall suggesting a faster differentiation pace (Brown and Lamartiniere 1995). Treatment of postpubertal rodents with steroids or human chorionic gonadotropin increases differentiation of the MG in a manner thought to mimic development during pregnancy (Russo and Russo 2004b; Sivaraman et al. 1998).

*Phytoestrogens.* Effects of treatment with phytoestrogens such as genistein are similar to those observed after estrogen receptor agonist exposure; perinatal exposure can lead to increased proliferation, and peripubertal exposure can lead to accelerated differentiation (reviewed by Warri et al. 2008). For example, Hilakivi-Clarke et al. (1998) and Padilla-Banks et al. (2006) showed increased TEBs after perinatal genistein treatment, and Cotroneo et al. (2002) showed accelerated development in MG after prepubertal exposure, as indicated by increased TEBs and ductal branching at an early time point, compared with untreated animals. After gestational and lactational genistein exposure, You et al. (2002) observed enhanced glandular differentiation at weaning, and males were more sensitive to the effect than females. Male rats in a multigenerational genistein feeding study also showed ductal hyperplasia at PND50, a surprisingly early life stage for these effects (Latendresse et al. 2009). Effects on MG development have also been observed after perinatal exposure to other phytoestrogens, including zearalanone and resveratrol, and to flaxseed [see Supplemental Material, Table 1 (doi:10.1289/ehp.1002864)].

*Environmental chemicals.* Altered MG development after perinatal exposure has also been observed for numerous EDCs, including atrazine, bisphenol A (BPA), dibutylphthalate, dioxin, methoxychlor, nonylphenol, polybrominated diphenyl ethers, and PFOA. Changes include delayed MG development, ductal hyperplasia, alveolar hypoplasia, reduced apoptosis in TEBs, altered gene or protein expression, increased or decreased numbers of terminal ducts or lobules, and accelerated alveolar differentiation [see Supplemental Material, Table 1 (doi:10.1289/ehp.1002864)], as well as increased MG tumors after carcinogen challenge (Brown et al. 1998; Durando et al. 2007; Jenkins et al. 2007). In addition, late-gestational treatment with Ziracin, a candidate antibacterial drug, induced hypoplasia (ducts without any acinar development) in rats (Poulet et al. 2005).

*Critical exposure windows and reversibility.* Studies of the ubiquitous industrial pollutant dioxin and the high-use herbicide atrazine have investigated critical periods of exposure associated with MG effects. Atrazine delayed MG development when exposure occurred around GD17–19 but had less of an effect after earlier 3-day windows, and dioxin exposure at GD15, but not after GD19, led to MG underdevelopment (Fenton et al. 2002; Rayner et al. 2005). More recent studies on the industrial surfactant PFOA (White et al. 2009) demonstrate a similar critical period. The heightened sensitivity during this time period is attributed to the formation of the mammary bud and initial branching that occurs during late pregnancy. As discussed above, exposure timing and dose influence the pattern of MG changes (Warri et al. 2008).

Although numerous studies have shown persistent effects on the MG, few have evaluated whether the changes could be reversible. For example, *in utero* exposure to dioxin, Ziracin, PFOA, or BPA led to permanent changes in the adult MG (Fenton et al. 2002; Poulet et al. 2005; Vandenberg et al. 2007; White et al. 2009). In contrast, effects of genistein and ethinyl estradiol in male MG appeared to reverse after treatment withdrawal (Latendresse et al. 2009). It is unclear whether the persistence of alterations reflects the biological half-life and lipophilicity of the chemical or epigenetic changes, and this may differ by compound.

*Mechanisms.* It is striking that MG developmental changes have been observed after exposure to diverse agents, including estrogens, androgens, antiandrogens, thyroid-active chemicals, and aryl hydrocarbon receptor agonists. Data do not indicate a similar mode of action for atrazine, but PFOA, brominated diphenyl ethers, and dioxin all have been shown to induce a phenotypically similar response of delayed MG development after neonatal exposures [see Supplemental Material, Table 1 (doi:10.1289/ehp.1002864)]. Novel mechanisms continue to be discovered. In a recent study, Doherty et al. (2010) found that *in utero* exposure of mice to DES or BPA increased protein expression and functional activity of the histone methyltransferase enhancer of zeste homolog 2 (EZH2) in the MG. EZH2 has been linked to breast cancer risk and epigenetic regulation of tumorigenesis. Its up-regulation is a potential mechanism through which *in utero* exposure to these chemicals may produce epigenetic changes leading to increased breast cancer risk (Doherty et al. 2010).

*Sex differences.* The few studies that have evaluated effects on male MG have indicated that male rats could be more sensitive. For example, one study found altered MG in males, but not females, treated with methoxyclor during gestation (You et al. 2002), and MG effects of genistein and ethinyl estradiol have been reported in males at lower doses than in females (Delclos et al. 2001; Latendresse et al. 2009). Study of sex differences in responsiveness can provide information about mechanisms of action for the test agents. Although male mice lack mammary epithelia, there are transgenic mouse models in which mammary epithelial growth can be induced in males (Li et al. 2002). Mouse models are needed to study some chemicals, such as PFOA, whose pharmacokinetics in mice and humans are most similar.

The Organisation for Economic Co-operation and Development (OECD) guidelines for subchronic oral toxicity testing (OECD 2008) include evaluation of the male, but not female, MG as an optional end point. In some studies using these guidelines, the male MG appears to be among the most sensitive end points evaluated (Okazaki et al. 2001), and at least one such study has found it to be the most sensitive end point in males (Andrews et al. 2002).

## Consequences of Altered Mammary Gland Development

Developmental exposures to certain EDCs can lead to MG developmental effects, lactational deficits, or cancer, but little is known about the relationships between the developmental and adult end points. The morphological changes in MG development, particularly effects on TEBs, suggest the potential for functional outcomes such as lactational insufficiency, altered pubertal timing, preneoplasia, or increased susceptibility to carcinogens (Fenton 2006). Table 1 and Supplemental Material, Table 2 (doi:10.1289/ehp.1002864) show these types of effects occurring across rodent and human studies for selected compounds for which there are data. However, there are data gaps regarding the relationships among the various MG outcomes because *a*) only a handful of chemicals have been studied; *b*) there has not been a standard procedure to assess MG developmental changes; *c*) MG assessment in multigenerational studies has been limited; and *d*) few studies include full assessment of dose response.

**Table 2:** MG as a sensitive end point of endocrine disruption after developmental exposures in rodents.

Compound	Study	Species, exposure timing	MG effect type*b*	MG effect LOEL	Basis for inclusion*c*
Females										
BPA		Jenkins et al. 2009		Rat, postnatal (lactation)		Proliferation*d*		250 μg/kg/day		No effects on age of VO, body weight, serum progesterone, or serum estradiol at 250 μg/kg/day (highest dose tested)
		Murray et al. 2007		Rat, prenatal		Hyperplasia*e*		2.5 μg/kg/day		No effects on body weight, age of VO, litter size, or sex ratio at this or higher doses (2.5–1,000 μg/kg/day).
		Muñoz-de-Toro et al. 2005		Mouse, perinatal		Morphology*f*, proliferation*d*		25 ng/kg/day		No effects on plasma estradiol at first proestrus at this or higher dose (250 ng/kg/day)
DDT		Brown and Lamartiniere 1995		Rat, peripubertal		Proliferation*d*		50 ng/kg/day		Single-dose study; no effects on body weight or uterine-ovarian weight
Genistein		Fritz et al. 1998		Rat, prenatal and postnatal		Morphology*f*		25 mg/kg/day		No effects on body weight, uterine weight, AGD, estrous cyclicity, or age at VO at this or higher dose (250 mg/kg/day)
		Padilla-Banks et al. 2006		Mouse, neonatal		Morphology*f*		0.5 mg/kg/day		Effects on ability to deliver live pups and estrous cyclicity at 50 mg/kg/day (but not at either 0.5 or 5 mg/kg/day)
Males										
Genistein		Delclos et al. 2001		Rat, prenatal and postnatal		Size*g*; hyperplasia*e*		25 ppm		Effects on ventral prostate weight, pituitary weight, age of eye opening and age of ear unfolding at 1,250 ppm
Abbreviations: AGD, anogenital distance; LOEL, lowest observed effect level. **a**For inclusion, a study must have assessed other end points in addition to MG; findings are based on statistically significant effects observed. **b**All effects are relative to negative controls; effects on protein or gene expression are omitted. For more detail, see Supplemental Material, Table 1 (doi:10.1289/ehp.1002864). **c**See articles for further study methods and results. **d**Changes in markers of proliferation/mitotic activity (e.g., cell cycle marker proliferating cell nuclear antigen, cell number). **e**Changes in numbers or sizes of hyperplastic structures. **f**Changes in numbers/ratios of structures, branching, and so on for given developmental stage. **g**Changes in the area or weight of gland.										

*Carcinogenesis.* Reported changes in patterns of breast development in U.S. girls (reviewed by Euling et al. 2008) raise concerns about whether earlier onset of breast development is associated with breast cancer or other adult diseases, because earlier menarche is an established risk factor for breast cancer (Kelsey et al. 1993). Furthermore, studies in humans and rodent models demonstrate that hormonal factors that affect MG development also influence susceptibility to carcinogens.

Hormonal factors alter susceptibility to carcinogens. Ovarian, pituitary, and placental hormones, which vary by life stage and with pregnancy events, are important determinants of breast cancer susceptibility in humans and rodents (Russo and Russo 2004b). In both mice and rats treated with chemical carcinogens, hormone withdrawal (ovariectomy) inhibits tumor development, whereas hormone supplementation increases the incidence of adenocarcinoma. In humans, removal of ovaries by 35 years of age dramatically reduces breast cancer risk (Eisen et al. 2005; Trichopoulos et al. 1972), and antiestrogens are effective in breast cancer treatment and chemoprevention (Vogel et al. 2010).

Susceptibility to carcinogens depends on life stage. The influence of life stage on susceptibility to carcinogen exposure has been demonstrated in rats and humans. For example, ionizing radiation is maximally potent as a human breast carcinogen when exposure occurs during childhood or adolescence (Henderson et al. 2010; Land 1995); this observation is consistent with findings in rodents (Imaoka et al. 2009). The increased tumor response from carcinogen exposure early in life is attributed to the presence of proliferating and undifferentiated structures such as TEBs, which are present during the pubertal mammary epithelial expansion and display elevated DNA synthesis compared with other MG structures (Kleinberg et al. 2009). TEBs are considered the most vulnerable MG target structure for carcinogen exposure (Medina 2007; Russo and Russo 1996). In animals and humans, tumor response from carcinogen exposure is highest when exposure occurs during adolescence, when TEBs are still abundant (Henderson et al. 2010; Imaoka et al. 2009; Land 1995; Russo and Russo 2004b). As a result, there is concern that exposures to xenobiotics that increase the number or longevity of proliferating TEBs might increase susceptibility to breast cancer (Birnbaum and Fenton 2003; Fenton 2006). After the pubertal growth spurt and throughout adult life, it is the terminal ductal structures that give rise to breast cancers (Kleinberg et al. 2009; Medina 2007; Russo and Russo 2004b). During pregnancy, differentiation of terminal structures increases, and this differentiation has been hypothesized to account for lower MG sensitivity to carcinogens postpregnancy (Russo and Russo 2004b).

Early life exposures to (noncarcinogenic) chemicals may affect response to carcinogens in later life. Experimental models involving carcinogen challenge have been used widely to demonstrate that hormones and growth factors influence MG development, differentiation, and carcinogenesis; these models could readily be extended to evaluate increased cancer risk from early life environmental exposures. These models have been used, for example, to investigate potential chemopreventive agents that accelerate MG differentiation (e.g., by mimicking pregnancy hormones) and decrease tumor susceptibility (Cotroneo et al. 2002; Kleinberg et al. 2009; Russo and Russo 1996). Rodent models used in these studies include dimethylbenz[*a*]anthracene (DMBA) and nitrosomethylurea (NMU) challenge in rats and mice, and the mouse mammary tumor virus (MMTV) model. More recently, genetically modified mouse models have been used to study mammary tumors that are comparable with human breast tumors in their latency, histotypes, and endocrine responsiveness (Cardiff et al. 2000; Kamiya et al. 1995; Medina 2007; Russo and Russo 2004b; Thompson and Singh 2000).

In rodents, early life exposure to hormonally active agents affects MG tumor formation in carcinogen-challenge models. For example, neonatal estrogen (or androgen) treatment of mice (MMTV model) or rats (DMBA model) induced MG developmental changes and increased tumors (Lopez et al. 1988; Mori et al. 1976, 1979). In addition, early life exposures to genistein (Hilakivi-Clarke et al. 1998, 1999), alcohol (Hilakivi-Clarke et al. 2004), dioxin (Brown et al. 1998; Desaulniers et al. 2001; Jenkins et al. 2007), and oral BPA (Jenkins et al. 2009) caused increased MG tumor multiplicity and decreased latency after DMBA challenge at PND50. These effects were accompanied by altered MG development observed in whole mounts (genistein, alcohol, dioxin) or altered protein expression (BPA). Lifetime exposures (beginning prenatally) to genistein, ethinyl estradiol, and BPA have been reported to alter MG development and increase incidence of preneoplastic lesions in the MG, with a stronger effect in early adulthood than at 2 years of age, when MG histopathology is typically performed (Latendresse et al. 2009; Murray et al. 2007; Vandenberg et al. 2008). Short-term genistein treatment during the peripubertal period reduces MG tumors after carcinogen challenge, whereas perinatal or lifetime exposure seems to increase them, although studies are not consistent (reviewed by Warri et al. 2008). Similarly, both gestational (Brown et al. 1998) and prepubertal (Desaulniers et al. 2001) dioxin exposure caused increased MG tumors after carcinogen challenge, whereas later life exposure decreased spontaneous MG tumors (Kociba et al. 1978).

In humans, maternal factors that affect the fetal hormone environment also appear to affect later breast cancer risk in daughters, possibly by imprinting the developing MG, thereby altering future tissue responsiveness to hormonal stimulation (e.g., altering estrogen receptor levels or sensitivity) or to genotoxic insult (e.g., by increasing cell proliferation or diminishing differentiation). The hypothesis that *in utero* endocrine-related factors influence breast cancer risk of a daughter is supported by epidemiology studies that have found *a*) preeclampsia associated with reduced breast cancer risk in offspring and *b*) high birth weight correlated with higher breast cancer risk (Hoover and Troisi 2001; Troisi et al. 2007; Xue and Michels 2007). In addition, there is some evidence that *in utero* exposure to DES is associated with higher breast cancer risk in women [Palmer et al. (2006); however, Verloop et al. (2010) did not find an association] and with increased MG tumor incidence in rats (Rothschild et al. 1987). Furthermore, the single epidemiologic study of the EDC dichlorodiphenyltrichloroethane (DDT) that used prospective measures of adolescent/young adult exposure in relation to breast cancer risk (Cohn et al. 2007) found significant associations, whereas many studies in which DDT or its metabolite dichlorodiphenyldichloroethylene (DDE) were measured in older women did not observe an association with breast cancer.

Pregnancy is another critical window corresponding to a time of extensive MG proliferation and differentiation. DES exposure in pregnant women has been associated with increased breast cancer risk in the mother as well as her daughter (Titus-Ernstoff et al. 2001). A study of DMBA-challenged rats fed a high-fat diet during pregnancy showed an increase in circulating estrogen during pregnancy and increased mammary tumors (Hilakivi-Clarke et al. 1996).

*Lactation.* The American Association of Pediatrics (AAP) recommends that all infants receive breast milk during the first 6 months (AAP 1997) and, further, that they are fed breast milk exclusively during this time (AAP 2005), because of the numerous demonstrated benefits of breast-feeding. Although data are limited, reports estimate that 3–6 million mothers are unable to produce milk or have difficulty breast-feeding each year (Lew et al. 2009). The reasons for this remain unclear, especially given that lactation insufficiency can be the result of psychosocial as well as biological factors. However, environmental chemicals are one candidate explanation for inability to initiate and/or sustain breast-feeding (Neville and Walsh 1995).

Impaired lactation may be associated with altered MG development (decreased or unresponsive breast tissue) and/or endocrine disruption (improper hormonal support for lactation). Critical windows include pregnancy and lactation as well as puberty and the prenatal/ perinatal period. As such, exposure to an EDC during pregnancy has the potential to disrupt lactation in the mother and the daughter. In human studies, strong early findings of associations between serum DDE and shortened lactation in two populations have been only partly replicated, and few other agents have been studied (Cupul-Uicab et al. 2008; Gladen and Rogan 1995; Rogan et al. 1987). In rodents, impaired lactation has been observed in conjunction with altered MG development in one or more generations after gestational exposure to dioxin (Vorderstrasse et al. 2004), PFOA (White et al. 2007, 2009), atrazine (Rayner et al. 2005), BPA (California Office of Environmental Health Hazard Assessment 2009; Matsumoto et al. 2004), genistein [National Toxicology Program (NTP) 2008], and the candidate pharmaceutical Ziracin (Poulet et al. 2005). For example, atrazine fed to rats during gestation induced MG developmental changes in offspring, characterized by stunted development, and when these rats were bred, their offspring (second-generation) had significantly reduced weight gain, suggesting insufficient milk production (Rayner et al. 2005). In an example of effects on lactation in the dam, exposure of pregnant mice to PFOA decreased pup weight and survival, diminished differentiation/growth of dam MG, and induced some alterations in gene expression for milk proteins, which taken together suggest effects on lactation in the exposed dams (White et al. 2007, 2009).

Treatment during pregnancy has the potential to affect lactation in both the dam and offspring. Impaired lactation in the dams is typically identified because of decreased pup weight or survival, and impaired lactation in the offspring can be determined only in multigenerational studies where offspring are followed through successful reproduction and lactation (Makris 2011). The rodent models and assessment methods used in guideline studies are not adequate for identifying effects on lactation because the surrogate markers of pup weight and postnatal survival are not sensitive or specific indicators of impaired lactation (Makris 2011).

## Human Health Risk Assessment Issues

*MG assessment in chemical test guidelines.* MG development can be affected after early exposure to EDCs in rodents. However, few guideline studies for testing environmental chemicals include prenatal or early life dosing, and MG end points are limited primarily to indirect or surrogate observations during lactation and to clinical and pathological evaluation of adult mammary tissue (Makris 2011). For example, the standard 2-year rodent cancer bioassay, initiating treatment in young adult animals, is likely to be less sensitive to carcinogens than if developmental exposures were used, and it cannot provide information on altered susceptibility to carcinogens induced by early life exposures affecting MG development (Hovey et al. 2002; Medina 2007; Rudel et al. 2007; Russo and Russo 2004b; Singh et al. 2000; Thayer and Foster 2007).

To strengthen MG assessment and chemical testing, it is a priority to enhance histopathological evaluation of MG development (e.g., using longitudinal rather than transverse sectioning so that a larger tissue plane is evaluated), increase attention to evaluation of male MG tissue, and incorporate early life exposures in rodent subchronic and chronic/carcinogenicity studies. Consistent with these recommendations, the NTP has begun including gestational and lactational dosing in rats assigned to subchronic and carcinogenicity studies and is taking steps to include early life male and female MG whole-mount preparations and longitudinal MG sectioning in reproductive assessment and cancer studies (NTP 2010; Thayer and Foster 2007). Use of these expanded protocols will facilitate linking altered MG development with later life outcomes.

As a potential addition to some toxicity test guidelines, MG whole-mount assessment can demonstrate morphological changes in development and differentiation and define the temporal and spatial progression of epithelial development. Another important reason to include MG assessments in screening-level toxicology studies is to ensure that MG effects are identified and can be evaluated in more comprehensive studies. Specifically, data generated using whole mounts may be used to trigger further assessment, such as: *a*) sectioning tissue blocks, *b*) evaluating subsequent (e.g., F_1_) generations for lactational impairment, *c*) maintaining a population longer on study for spontaneous neoplasia evaluation, or *d*) evaluating altered tumor susceptibility using a carcinogen-challenge protocol. Furthermore, a whole mount may be the only indication of abnormal development in the male MG, which is sensitive to very low doses in some studies (Delclos et al. 2001; Latendresse et al. 2009). However, currently there are no standardized whole-mount procedures, and consideration of these data in chemical risk assessment has been limited. The OECD test guideline for an extended one-generation reproductive toxicity study (OECD 2010) could be revised to include assessment of MG development using whole mounts and/or more thorough histopathology (Hvid et al. 2010). In addition, MG assessment of males and females could be added to the U.S. EPA Endocrine Disruptor Screening Program (EDSP) pubertal development protocols (U.S. EPA 2009a, 2009b). MG developmental assessment could also be extended to include females in the OECD Test Guideline 407 pubertal protocol (OECD 2008).

Adding MG whole-mount procedures to EDSP or OECD test guidelines has raised concerns that *a*) these assays could be redundant to endocrine-sensitive end points assessed [e.g., anogenital distance, timing of vaginal opening (VO), circulating hormones, and estrous cyclicity], and *b*) that the procedure is too difficult to be consistently executed across laboratories. However, in some cases MG effects have been observed at lower doses than other EDC outcomes (Table 2), and there is concern that EDSP assays, which identify chemicals affecting estrogen, androgen, or thyroid activities, may not be sensitive to the many mechanisms that can affect breast development. It is reasonable, therefore, to include the MG whole mount in screening studies, at least on a provisional basis, to see if the information gathered is redundant or unique.

*Human relevance of rodent models.* Rodent models have been widely used to characterize the influence of susceptibility factors (e.g., ovarian, pituitary, and placental hormones; life-stage and reproductive events) on malignant transformation of the MG, and parallels between rodent and human MG structures and pathologies have been enumerated (Medina 2007; Russo and Russo 2004b; Singh et al. 2000). Although a few findings in the context of chemicals testing have generated concern about human relevance (reviewed by Rudel et al. 2007), an extensive body of breast cancer research demonstrates similarities between rodent and human MG development and carcinogenesis. These studies indicate that rodent mammary tumors mimic the diversity of human breast cancers with respect to important initiation processes, histopathology, hormone dependence, and host–target cell interactions (Boylan and Calhoon 1983; Imaoka et al. 2009; Medina 2007; Rudland et al. 1998; Russo et al. 2000; Russo and Russo 1993, 2004a; Singh et al. 2000). In general, research indicates greater cross-species similarities for MG development and cancer than for human menstrual and rodent estrous cyclicity or for human puberty and rodent VO—end points currently included in many EDC test protocols (U.S. EPA 2011).

An expert panel on MG tumors concluded that existing rodent models are useful as screening tools for identifying potential breast carcinogens (Thayer and Foster 2007). Further, the majority of chemicals that are positive for mammary tumors in the rodent cancer bioassay have some evidence of genotoxicity and many are multisite carcinogens, supporting relevance to humans (Rudel et al. 2007). Although there are many similarities in the hormonal control of lactation across species, less is known about the utility of the rodent as a model for predicting chemical effects on human lactation. In any case, many risk assessment guidelines operate on the principle that animal effects are considered relevant to humans in the absence of data to indicate otherwise (U.S. EPA 1991, 1996, 2005).

A related issue is the consideration of carcinogen-challenge models as indicators of altered carcinogen susceptibility. DMBA and NMU are primary breast-specific carcinogens that have been widely used in experiments designed to assess the alteration of the tumor response by hormones or other factors (Kamiya et al. 1995; Medina 2007; Singh et al. 2000). Despite the long-standing use of such carcinogen challenge experiments to assess effects of hormonal or developmental alterations on tumor susceptibility, the protocols are not common in chemical toxicity assessment. Risk assessors have not considered data from carcinogen challenge experiments because of concerns about the protocol representing a chemical mixture study and about the presumed lack of relevance of DMBA or NMU exposure to humans. However, a number of consistent findings of increased susceptibility have been observed in human and rodent studies across multiple MG end points for endogenous hormonal factors, DES, genistein, and dioxin, among others (Table 1). Models that consider the interactive effects of endogenous hormones and carcinogenic factors across multiple life stages are likely to be more relevant to human health than those with simpler design, because they better reflect the human experience.

*Relative sensitivity of MG effects.* A limited set of studies provide evidence that MG alterations may be more sensitive to some EDCs than are other hormonally responsive end points (Table 2). To precisely determine the relative sensitivity of EDC effects requires studies that include MG as part of a larger set of endocrine-sensitive end points. Of the studies that simultaneously evaluated MG morphology and at least one other EDC-sensitive end point after developmental dosing, a subset has detected effects on the MG at dose levels or during exposure periods that did not elicit observable changes in other end points.

*Adversity of MG developmental changes.* Hormonal factors either increase or decrease MG tumor susceptibility, and both transient and permanent effects have been observed on MG development. This raises the question of what types of alterations to MG development should be considered adverse. In the context of regulatory evaluation of chemicals, one point of view is that MG developmental changes reflect altered growth and development, effects considered adverse by the U.S. EPA Developmental Toxicity Risk Assessment Guidelines (U.S. EPA 1991). For comparison, there is also controversy in the risk assessment community about whether other common markers of altered pubertal timing (e.g., VO, preputial separation) have human relevance. These end points have nevertheless been considered adverse, as they are responsive to endogenous sex steroids, which are important regulators of sexual development conserved across mammalian species. By this reasoning, altered MG growth and development, which is known to have human relevance, should be considered adverse as well. The question of adversity was discussed by experts gathered at the workshop in the context of risk assessment. In spite of the outstanding questions, the majority perspectives among experts advance the view that MG development and subsequent effects represent a public health outcome of concern and are a priority for future research and assessment. Priority questions, current views, and outstanding issues for risk assessment are summarized in Table 3.

**Table 3:** Priority questions, current views, and issues for improving risk assessment for MG effects.

Priority question for risk assessment application	Current views	Outstanding issues
Are the rat and mouse adequate models for human MG development?		Current knowledge suggests that the rat and mouse are reasonable surrogates.		Lack of information about human pubertal development; mechanisms may differ among species.
What is the sensitivity of MG developmental effects?		*In utero* exposure in some studies leads to developmental effects at doses similar to or lower than other developmental and reproductive end points.		Few EDC studies assess both MG development and another sensitive end point of ED; there is a lack of human data to address dose response and a lack of standardized MG development protocol and assessment criteria.
Are MG developmental changes adverse?		These changes in MG are considered adverse because they represent alterations in growth and development*b* and may be a risk factor for lactation and/or cancer outcomes.		Varied definitions of “adversity,” depending on scientific discipline and context.
**a**Based on the majority viewpoint of the experts at the Mammary Gland Evaluation and Risk Assessment Workshop. **b**According to guidance from the U.S. EPA (1991, 1996).				

An important question is whether MG developmental alterations are plausibly related to increased tumor susceptibility by *a*) epigenetic imprinting of tissue, *b*) alteration of stem cell populations, or *c*) increased number or ontological duration of TEBs or other structures known to be more vulnerable to carcinogens. Some experts suggest that such agents should themselves be considered carcinogens. Indeed, the International Agency for Research on Cancer (IARC) deems an agent carcinogenic if it is “capable of increasing the incidence of malignant neoplasms, reducing their latency, or increasing their severity or multiplicity” (IARC 2006). The U.S. EPA defines an effect as adverse if it “reduces the organism’s ability to respond to an additional environmental challenge” (U.S. EPA 2010). Applying these definitions, compounds that cause cancer, either alone or in combination with other factors at a variety of points in a biological chain of events leading to tumor formation, may reasonably be considered carcinogens, including chemicals that increase susceptibility to cancer. Even if such agents are not designated as carcinogens, their profound impacts should encourage the risk assessment community to consider the increase in cancer susceptibility as an adverse effect and therefore to characterize doses required to elicit the effect. In any case, applying this approach to risk assessment requires a better understanding of the relationship between altered MG development and carcinogen susceptibility.

## Conclusions and Research Recommendations

Research demonstrates many similarities between humans and rodents in normal and perturbed MG development and carcinogenesis. In both humans and rodents, developmental exposure to hormones affects MG development and carcinogen susceptibility, and these findings are the basis for ongoing research to identify chemopreventative agents in humans and to determine how EDCs may alter breast cancer risk, pubertal timing, or lactation. EDCs with diverse mechanisms of action, including many not considered primarily estrogenic, alter MG development in rodents. In some cases, altered MG development can be the most sensitive endocrine end point.

The lack of consistent methods for evaluating and reporting MG changes makes it difficult to compare findings across studies, hindering consideration of MG developmental effects in risk assessment. Continued progress will require consistent approaches across laboratories, along with a discussion of unique findings and unanticipated effects. In addition, the relationships between altered development and effects on lactation or carcinogenesis are still being defined. Addressing these research needs [detailed in Supplemental Material (doi:10.1289/ehp.1002864)] is a priority, and enhanced chemical testing and risk assessment are needed to characterize these effects.

Major research initiatives under way include The National Children’s Study, which has a number of EDC hypotheses proposed for testing in its longitudinal study (Lewin Group 2003), and the NIEHS Breast Cancer and the Environment Research Centers (NIEHS 2010), which have ongoing human studies focusing on the relationship between environmental exposures and age of breast development onset and which also support experimental animal research in this area. Research priorities identified at the workshop [provided in detail in Supplemental Material (doi:10.1289/ehp.1002864)] include further development and validation of the MG whole-mount protocol, research to establish the relationship between effects on MG development and later life outcomes, and issues relevant to use of these data in risk assessment.

## Supplemental Material

(352 KB) PDF
