# Supplementary material for: Environmental Exposures and Mammary Gland Development: State of the Science, Public Health Implications, and Research Recommendations
Source: Environ Health Perspect. 2011 Jun 22;119(8):1053–61. doi: 10.1289/ehp.1002864 (PMC3237346; doi:10.1289/ehp.1002864)
Supplement: (352 KB) PDF [file ehp.1002864.s001.pdf]

Supplemental Material for Environmental Health Perspectives

Environmental Exposures and Mammary Gland Development: State of the Science, Public Health Implications, and Research Recommendations

Rudel, Ruthann A.,[1*] Fenton, Suzanne E.,[2] Ackerman, Janet M.,[1] Euling, Susan Y.,[3] Makris, Susan L.[3]

[1]Silent Spring Institute, Newton, MA 02458
[2]NIEHS, National Toxicology Program, Research Triangle Park, NC 27709
[3]NCEA, US Environmental Protection Agency, 1200 Pennsylvania Ave., NW, Washington, DC 20460

*Address correspondence to Ruthann Rudel, Silent Spring Institute, 29 Crafts Street, Newton, MA 02458; phone 617-332-4288 x214; fax 617-332-4284; rudel@silentspring.org

Supplemental Material:

- Workshop participants and affiliations .... page 2
- Workshop findings: Research priorities and recommendations .... page 3
- Supplemental Material, Table 1 a-c: Studies Describing Altered Mammary Gland Development
  Description .... page 4
  a) females-in utero/perinatal exposure .... page 5
  b) females-peripubertal exposure .... page 11
  c) males .... page 13
- Supplemental Material, Table 2: Female Mammary Gland Outcomes After Developmental Exposure: rodent-human concordance for selected agents (fully cited version of Table 1, which appears in the article in a condensed form) .... page 14
- References .... page 16

Workshop participants and affiliations:

Mary Helen Barcellos Hoff, New York University
Janice Barlow, Zero Breast Cancer
Don Bergfelt, US EPA
Linda Birnbaum, NIEHS
Frank Biro, Cincinnati Children's Hospital Medical Center
Julie Boberg, Technical University of Denmark
Julia Brody, Silent Spring Institute
John Budroe, CA EPA OEHHA
Robert Cardiff, University of California Davis
Barbara Cohn, Child Health and Development Studies
Gwen Collman, NIEHS
Daniel Desaulnier, Health Canada
Caroline Dilworth, NIEHS
Jim Donald, CA EPA OEHHA
Elizabeth Doyle, US EPA, Office of Water
Susan Euling, US EPA NCEA
Sue Fenton, NIEHS
Laurence Fitzgerald, California Breast Cancer Research Program
James M. Ford, California Breast Cancer Research Program
Warren Foster, McMaster University
Michael Gould, University of Wisconsin
Janet Gray, Vassar College, Breast Cancer Fund
Sandra Haslam, Michigan State University
Ann Hernick, Breast Cancer Alliance of Greater Cincinnati
Leena Hilakivi-Clarke, Georgetown University
Russell Hovey, University of California Davis
Laura Kass, University of Litoral, Santa Fe, Argentina
Marion Kavanaugh-Lynch, California Breast Cancer Research Program
Susan Knadle, CA EPA OEHHA
Andreas Kortenkamp, School of Pharmacy, University of London
Larry Kushi, Kaiser Permanente
John Latendresse, National Center for Toxicological Research
Betina Lew, University of Rochester Medical Center
Anna Lowit, US EPA
Amal Mahfouz, US EPA
Susan Makris, US EPA NCEA
Elizabeth Maull, NIEHS
Katherine McKenzie, California Breast Cancer Research Program
Daniel Medina, Baylor College of Medicine
Rekha Mehta, Health Canada
Wynne Miller, US EPA
Monica Munoz-de-Toro, University of Litoral, Santa Fe, Argentina
Elizabeth Padilla-Banks, NIEHS
Laura Perovich, Silent Spring Institute
Susan Pinney, University of Cincinnati
Svetlana Popova, California Department of Public Health
Ahmed Raafat, National Institutes of Health
Leslie Reinlib, NIEHS
Jeanne Rizzo, Breast Cancer Fund
Gertraud Robinson, National Institute of Diabetes and Digestive and Kidney Diseases
Walter Rogan, NIEHS
Paul Rowsell, Health Canada
Ruthann Rudel, Silent Spring Institute
Jose Russo, Fox Chase Cancer Center
Irma Russo, Fox Chase Cancer Center
Martha Sandy, CA EPA OEHHA
Meg Schwarzman, University of California Berkley
Pam Shubat, Minnesota Health Department
Suzanne Snedeker, Cornell University Program on Breast Cancer and Environmental Risk Factors
Carlos Sonnenschein, Tufts University
Ana Soto, Tufts University
Jason Stanko, NIEHS
Claudia Thompson, NIEHS
Catherine Thomsen, California Breast Cancer Research Program
Beth Vorderstrasse, Washington State University
Anni Warri, Georgetown University/University of Turku (Finland)
Terri Wood, New Jersey Medical School Cancer Center
Lily Wu, CA EPA OEHHA
Mary Alice Yund, California Breast Cancer Research Program

Workshop findings: Research priorities and recommendations

Relationship between altered MG development and later life outcomes (lactation and cancer):

- Develop models for evaluating and quantifying altered susceptibility to carcinogens, and determine the features of altered MG development that are influential.
- Characterize the relationships between altered MG development and impaired lactation.
- Develop sensitive exposure measures for hormones and growth factors that regulate MG development, including total steroid hormone (estrogen and progesterone) levels/activity, tissue-specific (liver, ovary, MG) endogenous steroid hormone (estrogen and progesterone) levels/activity.
- Identify early biomarkers of MG cancer effects (e.g., hyperplasia, immunohistochemical markers, MG-specific gene markers, presence of nodules and bridging, changes in hormone levels, changes in hormone receptor levels, receptor sensitivity, effects on stromal-epithelial interactions) by utilizing banked MG tissue blocks, serum samples, and other stored tissues from longer term studies that observed MG tumors.
- Evaluate epigenetic changes in mammary epithelium and stroma following prenatal exposures to better understand early life programming.
- Evaluate the relationship between early life EDC exposure with later life outcomes in epidemiologic studies (e.g., lactational insufficiency, male and female breast cancer, male gynecomastia).
- Consider use of transgenic mouse models for specific human breast cancer phenotypes or further understanding of male breast cancer (e.g., Her2, P450 aromatase)

Chemical toxicity testing and risk assessment:

- Complete development and validation of MG whole mount protocol to support consistent reporting of effects on MG growth and development.
- Perform studies using the MG whole mount evaluation in parallel with other EDC-sensitive endpoints (e.g., AGD, cyclicity, VO) for more EDCs to assess relative sensitivity. Include assessment of male MG effects to provide insight on key mechanisms.
- Add MG whole mount assessment to toxicity test guidelines such as OECD extended one-generation reproductive toxicity and EPA pubertal protocols.
- Evaluate adequacy of rodent model for identifying exposures that lead to impaired lactation; develop additional more direct and sensitive endpoints for guideline studies.
- Further characterize normal human breast development, especially during puberty. Characterize normal development as well as perturbed development and carcinogenesis in animal models and humans. Characterize differences in susceptibility between animal models and humans and among humans.
- Describe interaction of genetics, stress, diet, endogenous hormones and exogenous chemicals as they influence MG development.
- Measure or estimate internal dose in MG studies to improve understanding of tissue distribution and pharmacokinetics of different EDCs known to affect MG development.

Supplemental Material Table 1 Description

Supplemental Material Table 1 is a systematic compilation of studies that have evaluated the effects of hormone, dietary, or chemical exposures during the prenatal, neonatal, or peripubertal periods on mammary gland development up to 10 weeks. It comprises 3 sub tables: part a describes experiments evaluating female mammary glands after prenatal/neonatal exposures (GD 0-weaning); part b describes experiments evaluating female mammary glands after peripubertal exposure (~PND 16-50), and part c describes experiments evaluating male mammary glands after prenatal, neonatal, or peripubertal exposure. This table includes several additional endocrine-sensitive endpoints that are commonly assessed, in order to indicate relative sensitivity. Some of the papers listed may have reported mammary gland or other effects after 10 weeks, or may have reported other endocrine effects that are not included in this table. Based on an extensive PubMed literature review and examination of the citations in these papers, we expect that this table is fairly comprehensive, although a few relevant studies may be missing.

Supplemental Material Table 1: Studies describing altered mammary gland development: a) females-in utero/perinatal exposure

Compound	Study	Animal	ROE	Dosing interval	Doses	MG effects	Pubertal timing[a]	Estrous cycle	AGD	Ovarian wt	Uterine wt	Serum E2	Corpora lutea
Endogenous or pharmaceutical hormones													
Diethylstilbestrol (DES)	Doherty et al. 2010	CD-1 mouse	IP	GD 9-26	0.01 mg/kg/d	Altered protein expression, increased histone methylation at PNW 6							
	Ninomiya et al. 2007	Sprague Dawley rat	SC	PND 0	0.0001 mg/kg/d	Increased TEBs at PND 50		NC					CL absent in some mice at PND 50
					0.001 mg/kg/d	" "		Altered					CL absent in some mice at PND 50
					0.01 mg/kg/d	Reduced TEBs at PND 50		Altered					CL absent in some mice at PND 50
					0.1 mg/kg/d	" "		Altered					CL absent in some mice at PND 50
	Hovey et al. 2005	BALB/c mouse	SC	PND 1	0.0125 mg/kg	Increased ductal outgrowth at PND 33							
	Nikaido et al. 2004	Crj:CD-1 mouse	SC	GD 15-18	0.0005 mg/kg/d	NC[b]	Early	NC					CL absent in some mice at 4 weeks
					0.01 mg/kg/d	NC	Early	Altered					" "[d]
	Fielden et al. 2002	B6D2F1 mouse	Gavage	GD 12- PND 20	0.0001 mg/kg/d	NC			NC				
					0.001 mg/kg/d	Increase in mammary length at PND 49			NC				
					0.01 mg/kg/d	" "			NC				
	Tomooka and Bern 1982	BALB/cCrgl mouse	SC	PND 1-5	0.0001 mg/kg/d	Reduced branching at PND 6 and PND 33							
					0.002 mg/kg/d	Reduced branching at PND 6							
17b-estradiol	Yin et al. 2006	Sprague Dawley rat	SC	PND 2, 4, 6	0.0001 mg/d	NC				NC	NC		
					0.01 mg/d	NC				NC	NC		
	Cabanes et al. 2004	Sprague Dawley rat	SC	PND 7-20	0.01 mg/d	Reduced TEBs at PNW 8	Early	NC			NC	NC	
	Hilakivi-Clarke et al. 1998	CD-1 mouse	SC	GD 15-20	0.02 mg/kg/d	Increased TEB density by PND 46	Early	NC				NC	
	Hilakivi Clarke et al. 1997b	Sprague Dawley rat	SC	GD 14-20	0.00002 mg/d	Increased cell density, TEBs at PNW 4; increased TEBs, reduced ABs by PNW 7	Early				NC	NC	
	Hilakivi Clarke et al 1997a	CD-1 mouse	SC	PND 0-2	0.002-0.004 mg/d	Increased TEBs PND 25, 50, reduced LAUs PND 50							
	Tomooka and Bern 1982	BALB/cCrgl mouse	SC	PND 1-5	0.005 mg/kg/d	Reduced branching at PND 6, increased branching at PND 33							
					0.02 mg/kg/d	" "							
	Warner 1976	BALB/cCrgl mouse	SC	PND 1-5	0.025 mg/d	NC							
					0.035 mg/d	Increased size, branching at PNW 5							
					0.07 mg/d	" "							

Supplemental Material Table 1: Studies describing altered mammary gland development: a) females-in utero/perinatal exposure

Compound	Study	Animal	ROE	Dosing interval	Doses	MG effects	Pubertal timing[a]	Estrous cycle	AGD	Ovarian wt	Uterine wt	Serum E2	Corpora lutea
ICI 182,780 (ER antagonist)	Hilakivi-Clarke et al. 1997a	CD-1 mouse	SC	PNW 0-2	0.02 mg/kg/d	Increased epithelial cell density at PND 41, increased TEBs, LAUs at PND 54							
MER-25 (ethamoxytriphetol), an antiestrogen	Jean-Faucher et al. 1977	Swiss mouse	SC	GD 13-18	5 mg/d	Delayed development of nipple, mammary cord and adjacent mesenchyme; generalized athelia at GD 19							
Tamoxifen	Hovey et al. 2005	BALB/c mouse	SC	PND 1	0.00125 mg/kg	NC							
					0.025 mg/kg	Reduced ductal outgrowth at PND 33							
	Hilakivi-Clarke et al. 1998	CD-1 mouse	SC	GD 15-20	0.002 mg/d	Increased epithelial cell area at PND 35, increased TEB density at PND 35, 46	Early	NC				NC	
Testosterone	Tomooka and Bern 1982	BALB/cCrgl mouse	SC	PND 1-5	0.005 mg/kg/d	Increased branching at PND 33							
					0.02 mg/kg/d	As above, and increased branching at PND 26							
5a-dihydro-testosterone	Tomooka and Bern 1982	BALB/cCrgl mouse	SC	PND 1-5	0.02 mg/kg/d	Increased ductal branching at PND 33							
5b-dihydro-testosterone	Tomooka and Bern 1982	BALB/cCrgl mouse	SC	PND 1-5	0.02 mg/kg/d	NC							
Transforming growth factor alpha	Hilakivi Clarke 1997a	CD-1 mouse	SC	PND 0-2	0.004 mg/d	TEBs increased at PND 25, 35, reduced by PND 50; LAUs increased at PND 35 and 50							
Dietary components													
Biochanin A	Yin et al. 2006	Sprague-Dawley rat	SC	PND 2, 4, 6	0.1 mg/d	NC				NC	NC		
					10 mg/d	Reduced TEB density at PND 35				NC	NC		
Flaxseed	Tan et al. 2004	Sprague-Dawley rat	Diet	PND 1-21	10%	Increased TEBs, TDs at PND 21; reduced TEBs at PND 50	NC	NC	NC	NC	NC		
(Secoisolari-ciresinol diglucoside)	Tan et al. 2004	Sprague-Dawley rat	Diet	PND 1-21	10%	Reduced TEBs at PND 50	NC	NC	NC	NC	NC		
Flaxseed	Ward et al. 2000	Rat	Diet	PND 1-21	10%	Reduced TEB density at PND 50							
				PND 1-50	10%	Reduced TEB density, increased AB density at PND 50							
(Secoisolari-ciresinol diglucoside)	Ward et al. 2000	Rat	Diet	PND 1-21	10%	Reduced TEB density, increased AB density at PND 50							
				PND 1-50	10%	Reduced TEB density, increased AB, lobule density at PND 50							
Flaxseed	Tou and Thompson 1999	Sprague-Dawley rat	Diet	GD 0-PND 21 or -PND 50	5%	Reduced TEB density at PND 50	Delayed	NC		NC		NC	
			Diet		10%	Reduced TEB density, increased AB density at PND 50	Early	Altered		Increased		Increased	
(Flaxseed oil)			Diet	GD 0-PND 21	1.82% (equivalent to 5% flaxseed)	NC	NC	NC		NC		NC	
(SDG)			Gavage		Equivalent to 5% flaxseed	Reduced TEB and AB density at PND 50	Delayed	NC		NC		NC	

Supplemental Material Table 1: Studies describing altered mammary gland development: a) females-in utero/perinatal exposure

Compound	Study	Animal	ROE	Dosing interval	Doses	MG effects	Pubertal timing[a]	Estrous cycle	AGD	Ovarian wt	Uterine wt	Serum E2	Corpora lutea
Genistein[c]	Hilakivi-Clarke et al. 2002	Sprague Dawley rat	Diet	GD 0-PND 0	150 ppm[d]	NC						NC	
					300 ppm[d]	Increased TEBs, reduced lobule density at PNW 8						Reduced PNW 8	
	Delclos et al. 2001	Sprague-Dawley rat (both sexes)	Diet	GD7-PND50	5 ppm	NC	NC		NC	NC	NC		NC
					25 ppm	NC	NC		NC	NC	NC		NC
					100 ppm	NC	NC		NC	NC	NC		NC
					250 ppm	Hyperplasia, increased proliferation at PND 50	NC		NC	NC	NC		NC
					625 ppm	As above, and increased TEBs at PND 50	NC		NC	NC	NC		NC
					1250 ppm	" "	NC		NC	NC	NC		Fewer and smaller
	Fritz et al. 1998	Sprague Dawley CD rat	Diet	GD 0-PND 21	25 ppm	Increased ratio of lobules:TEBs, fewer lobules I at PND 50	NC	NC	NC		NC		
					250 ppm	As above, and reduced TEBs, TDs at PND 21; reduced TEBs at PND 50	NC	NC	NC		NC		
	Padilla-Banks et al. 2006	CD-1 mouse	SC	PND 1-5	0.5 mg/kg/d	Increased ductal elongation at PNW 6		NC					
					5 mg/kg/d	Reduced branching at PNW 5, reduced TEBs at PNW 6		NC					
					50 mg/kg/d	As above, and reduced TEBs at PNW 5		Altered					
	Nikaido et al. 2004	Crj:CD-1 mouse	SC	GD 15-18	0.5 mg/kg/d	NC	Early	Altered					
					10 mg/kg/d	NC	Early	Altered					Corpora lutea absent in some mice at 4 weeks
	Fielden et al. 2002	B6D2F1 mouse	Gavage	GD 12-PND20	0.1 mg/kg/d	NC			NC				
					0.5 mg/kg/d	NC			NC				
					2.5 mg/kg/d	NC			NC				
					10 mg/kg/d	NC			NC				
	Hilakivi-Clarke et al. 1998	CD-1 mouse	SC	GD 15-20	0.02 mg/kg/d	Increased TEB density at PND 35, 46, increased epithelial cell area, reduced TD, AB density at PND 35	Early	NC				NC	
Polyunsaturated fatty acids (PUFA)	Hilakivi-Clarke et al. 2002	Sprague Dawley rat	Diet	GD 0-PND 0	16% calories from fat (n-6 PUFA)	[f]						[f]	
					16% calories from fat (n-3 PUFA)	[f]						[f]	
					39% calories from fat (n-6 PUFA)	Increased TEBs at PNW 3[f]						Serum E2 lower in PNW 8[f]	
					39% calories from fat (n-3 PUFA)	Reduced TEBs at PNW 8[f]						[f]	
	Hilakivi Clarke et al. 1997b	Sprague Dawley rat	Diet	GD 0-PND 0	43-46% calories from corn oil (high n-6 PUFA)[f]	Mammary fat pad area larger, TEBs and epithelial cells denser at PNW 4, 7, 11; ABs denser at PNW 11	Early	NC			NC	NC	

Supplemental Material Table 1: Studies describing altered mammary gland development: a) females-in utero/perinatal exposure

Compound	Study	Animal	ROE	Dosing interval	Doses	MG effects	Pubertal timing[a]	Estrous cycle	AGD	Ovarian wt	Uterine wt	Serum E2	Corpora lutea
Resveratrol	Nikaido et al. 2004	Crj:CD-1 mouse	SC	GD 15-18	0.5 mg/kg/d	NC	NC	Altered					
					10 mg/kg/d	NC	NC	Altered					Corpora lutea absent in some mice at 4 weeks
Zearalenone	Nikaido et al. 2004	Crj:CD-1 mouse	SC	GD 15-18	0.5 mg/kg/d	Accelerated alveolar differentiation at PNW 4	Early	Altered					
					10 mg/kg/d	As above, and retarded growth in mice lacking copora lutea	Early	Altered					Corpora lutea absent in some mice at 4 weeks
	Hilakivi-Clarke et al. 1998	CD-1 mouse	SC	GD 15-20	0.002 mg/kg/d	Increased TEB density at PND 35; increased epithelial cell, TD, AB density at PND 46	NC	Altered				NC postnatal	
Environmental chemicals													
Atrazine	Enoch 2007 (Atrazine metabolite mixture)	Long Evans rat	Gavage	GD 15-19	0.09 mg/kg/d (AMM)	Stunted growth pattern at PND 25, 40, 60	NC	NC		Increased	NC		
					0.87 mg/kg/d (AMM)	As above, and stunted growth pattern at PND 4	NC	NC		NC	NC		
					8.73 mg/kg/d (AMM)	Stunted growth pattern at PND 25, 33, 40, 60	NC	NC		NC	NC		
					100 mg/kg/d (atrazine)	As above, and stunted growth pattern at PND 4	NE[g]	Altered		NC	NC		
	Moon et al. 2007	Long Evans rat	Gavage	GD 15-19	100 mg/kg/d	Reduced distance between glands at PND 33				NC	NC		
	Rayner et al. 2005	Long Evans rat	Gavage	GD 13-19 (and 2-day periods within)	100 mg/kg/d	Delayed development, reduced MG size at PND 4-67 (effects seen after 2-day and 7-day dosing)	Delayed (only after dosing GD 13-19)	NC		NC	NC	NC	
	Rayner et al. 2004	Long Evans rat	Gavage	GD 15-19, cross fostered for lactation	100 mg/kg/d	Reduced size, immature ductal structures PND 4; sparse branching, delayed migration PND 22; reduced size, increased TEBs PND 40	NC	NC		NC	NC		
				lactation only (dams dosed GD 15-19)		As above, and reduced size, increased TEBs at PND 33, increased proliferation at PND 40	Delayed	NC		NC	NC		
				GD 15-19+ lactational exposure		" "	Delayed	NC		NC	NC		
Bisphenol A	Jenkins et al. 2009	Sprague Dawley rat	Gavage	PND 2-20	0.025 mg/kg/d	NC	NC	NC				NC	
					0.25 mg/kg/d	Increased cell proliferation, reduced apoptosis in TEBs at PND 50	NC	NC				NC	
	Moral et al. 2008	Sprague Dawley-CD rat	Gavage	GD 10-21	0.025 mg/kg/d	Altered gene expression							
					0.25 mg/kg/d	As above, and increased TDs at PND 21, 100, increased lobules 1 at PND 35							
	Durando et al. 2007	Wistar rat	SC	GD 8-23	0.025 mg/kg/d	Reduced apoptosis in parenchyma and stroma	Early		NC				
	Murray et al. 2007	Wistar Furth rat	SC	GD 9-PND1	0.0025 mg/kg/d	Increase in hyperplastic ducts	NC	NC					
					0.025 mg/kg/d	" "	NC	NC					
					0.25 mg/kg/d	As above, and cribiform-like structures	NC	NC					
					1 mg/kg/d	" "	NC	NC					

Supplemental Material Table 1: Studies describing altered mammary gland development: a) females-in utero/perinatal exposure

Compound	Study	Animal	ROE	Dosing interval	Doses	MG effects	Pubertal timing[a]	Estrous cycle	AGD	Ovarian wt	Uterine wt	Serum E2	Corpora lutea
Bisphenol A	Yin et al. 2006	Sprague Dawley rat	SC	PND 2, 4, 6	0.1 mg/d	NC				NC	NC		
					10 mg/d	Reduced TD and AB densities at PND 35				NC	NC		
	Doherty et al. 2010	CD-1 mouse	IP	GD 9-26	5 mg/kg/d	Altered protein expression, increased histone methylation							
	Vandenberg et al. 2006	CD-1 mouse	SC	GD 8-18	0.00025 mg/kg/d	Accelerated growth, loss of intrauterine positional effects at GD 18							
	Munoz-de-Toro et al. 2005	CD-1 mouse	SC	GD 9-PND 4	0.000025 mg/kg/d	Reduced apoptosis in TEBs, increased PR expression at PND 30						NC	
					0.00025 mg/kg/d	As above, and increased TEBs, reduced proliferation in the stroma at PND 30						NC	
	Nikaido et al. 2004	Crj:CD-1 mouse	SC	GD 15-18	0.5 mg/kg/d	NC	NC	Altered					CL absent in some mice at 4 weeks
					10 mg/kg/d	Accelerated alveolar differentiation at PNW 4 (in mice with corpora lutea)	Early	Altered					" "
	Markey et al. 2001	CD-1 mouse	SC	GD 9-PND 19	0.025 mg/kg/d	Altered pattern of cell division at PND 10							
					0.25 mg/kg/d	As above, and altered cell division at 1 month							
Butyl benzyl phthalate	Moral et al. 2007	Sprague Dawley CD rat	Gavage	PND 2-20	500 mg/kg/d	Changes in gene expression, cell proliferation pattern (no change in numbers of structures)	NC				Increased relative uterine weight PND 21, no change thereafter		
Dibutyl phthalate	Lee et al. 2004	CD(SD)IGS rat	Diet	GD 15-PND 21	20 ppm	Hypoplasia of alveolar bud at PND 21	NC	NC					
					200 ppm	" "	NC	NC					
					2000 ppm	NC	NC	NC					
					10000 ppm	Hypoplasia of alveolar bud at PND 21	NC	NC					
Dieldrin	Foster et al. 2008	BALB/c mice	Gavage	GD 7-weaning (weekly)	0.45 mg/kg/d	NC		NC					
					2.25 mg/kg/d	NC		NC					
					4.5 mg/kg/d	NC		NC					
Dioxin (TCDD)	Jenkins et al. 2007	Rat	Gavage	GD 15	0.001 mg/kg	NC							
					0.003 mg/kg	Altered protein expression (more proteins evaluated than at low dose)							
	Fenton et al. 2002	Long Evans rat	Gavage	GD 15	0.001 mg/kg	Delayed differentiation/development	Delayed	NC					
	Brown et al. 1998	Sprague-Dawley rat	Gavage	GD 15	0.001 mg/kg	Increased TEBs, reduced lobules II at PND 50	Delayed[h]	Altered[h]			NC		
Nonylphenol	Moon et al. 2007	Long Evans rat	Gavage	GD 15-19	10 mg/kg/d	NC				NC	NC		
					100 mg/kg/d	NC				NC	Increased		

Supplemental Material Table 1: Studies describing altered mammary gland development: a) females-in utero/perinatal exposure

Compound	Study	Animal	ROE	Dosing interval	Doses	MG effects	Pubertal timing[a]	Estrous cycle	AGD	Ovarian wt	Uterine wt	Serum E2	Corpora lutea
PBDEs (DE-71, a penta-brominated mixture)	Kodavanti et al. 2010	Long Evans rat	Gavage	GD 3-PND 21	1.7 mg/kg/d	NC	NC males[i]		NC males[i]				
					10.2 mg/kg/d	Reduced outgrowth, lateral branches, TEB development at PND 21	NC males[i]		NC males[i]				
					30.6 mg/kg/d	" "	Delayed in males[i]		NC males[i]				
PFOA	White et al. 2009	CD-1 mouse	Gavage	GD 1-17, lactation	3 mg/kg/d	Delayed ductal elongation and TEB appearance, reduced secondary and tertiary branching at PND 22, 42, 63							
					5 mg/kg/d	" "							
	White et al. 2007	CD-1 mouse	Gavage	GD 1-17	5 mg/kg/d	Reduced outgrowth and branching PND 10, 20							

AB: alveolar bud. AGD: anogenital distance. E2: estradiol. ER: estrogen receptor. FSH: follicle-stimulating hormone. GD: gestational day. LAU: lobulo-alveolar unit. LH: luteinizing hormone. PND:post-natal day. PNW: post-natal week. PR: progesterone receptor. ROE: route of administration (SC= subcutaneous; IP= intraperitoneal.) TD: terminal duct. TEB: terminal end bud. TSH: thyroid-stimulating hormone.

[a] pubertal timing: assessed as time of vaginal opening for females, preputial separation or testes descent for males.

[b] NC, no change: evaluated, but no significant changes observed. Changes may have been observed after PNW 10.

[c] due to the very large number of studies evaluating the mammary gland after genistein exposure, only a subset are presented in this table. See Warri et al. 2008 for review.

[d] all changes reported relative to control of 15 ppm.

[e] no control group; all results reported are differences from every other group.

[f] control is 12%-16% calories from corn oil (low n-6 PUFA).

[g] NE: not evaluated.

[h] unclear if significant.

[i] Pubertal timing and AGD measured only in males.

Supplemental Material Table 1: Studies describing altered mammary gland development: b) females-pubertal exposure

Compound	Study	Animal	ROE	Dosing interval	Doses	MG effects[a]	Pubertal timing	Estrous cycle	Ovarian wt	Uterine wt	Serum E2
Endogenous or pharmaceutical hormones											
DES	Odum et al. 1999a	Noble rat	SC	11 days starting at PNW 5-6	~0.076 mg/kg/d (0.01 mg/d)	Increased lobules I, II, III, reduced TDs, TEBs, cell cycle changes					
	Odum et al. 1999b	Alderley Park rat	SC	11 days starting at PNW 5-6	~0.055 mg/kg/d (0.01 mg/d)	Increased lobules II, III				NC	
			Gavage		0.075 mg/kg/d	Increased lobules I, II, III, reduced TDs, TEBs				NC	
	Colerangle and Roy 1997	Noble rat	SC	11 days starting at PNW 4-5	0.1 mg/kg/d	Increased proliferation					
	Colerangle and Roy 1996	Noble rat	SC	11 days starting at PNW 4-5	0.1 mg/kg/d	Increased # cells, proliferation					
	Brown and Lamartiniere 1995	Sprague Dawley rat	SC	PND 23, 25, 27, 29	0.05 mg/kg/d	Reduced TEBs, TDs, ABs, increased lobules, increased cell proliferation in terminal structures			Increased	Increased	
Estradiol benzoate (E2)	Cotroneo et al. 2002	Sprague Dawley rat	SC	PND 16, 18, 20	0.0005 mg/kg/d	Increased TEBs, ductal branching, proliferation; reduced TDs				Increased	
Dietary components											
Conjugated linoleic acid	Ip et al. 1999	Sprague Dawley rat	Diet	PND 23-55	0.8% : high-CLA butter fat	Reduced MG epithelium density, branching, TEB density, cell division in					
					0.8%: control butter fat + Matrya CLA	" "					
					0.8%: control butter fat + Nu-Chek CLA	" "					
Flaxseed	Tou and Thompson 1999	Sprague Dawley rat	Diet	PND 21-50	5%	NC		NC	NC		NC
					10%	NC		NC	NC		NC
Genistein	Santell et al. 1997	Sprague Dawley rat	Diet	PND 31-44	375 ppm	NC				NC	
					750 ppm	NC				NC	
	Murril et al. 1996	Sprague Dawley rat	SC	PND 16, 18, 20	500 mg/kg/dose	Rapid development of gland, yielding more differentiated structures, fewer undifferentiated by PND 50	Early	Altered			
	Santell et al. 1997	Sprague Dawley rat	Diet	PND 31-44	375 ppm	NC				NC	
					750 ppm	NC				NC	
	Brown and Lamartiniere 1995	Sprague Dawley rat	SC	PND 23, 25, 27, 29	0.05 mg/kg/d	Increased lobules, increased cell proliferation in terminal structures, increased gland size			Increased	Increased	

Supplemental Material Table 1: Studies describing altered mammary gland development: b) females-pubertal exposure

Compound	Study	Animal	ROE	Dosing interval	Doses	MG effects[a]	Pubertal timing	Estrous cycle	Ovarian wt	Uterine wt	Serum E2
Environmental chemicals											
Bisphenol A	Colerangle and Roy 1997	Noble rat	SC	11 days starting at PNW 5-6	0.1 mg/kg/d	Increased lobules I, II, number of cells, proliferation					
					54 mg/kg/d	As above, and increased lobules III, TEBs					
DDT (o-p)	Brown and Lamartiniere 1995	Sprague Dawley rat	SC	PND 23, 25, 27, 29	0.05 mg/kg/d	Increased cell proliferation in terminal structures			NC	NC	
Dioxin (TCDD)	Brown and Lamartiniere 1995	Sprague Dawley rat	Gavage	PND 25, 27, 29, 31	0.0000025 mg/kg/d	Reduced TEBs, reduced cell proliferation in TDs, lobules, reduced gland size			Reduced	Reduced	
Nonylphenol	Colerangle and Roy 1996	Noble Rat	SC	11 days starting at PNW 5-6	0.01 mg/d	Increased cell proliferation and lobular development, reduced TEBs, increased lobules II, III					
					7.12 mg/d	Increased cell proliferation and lobular development; reduced TDs, increased lobules I, II, III					
	Odum et al. 1999a	Noble Rat	SC	11 days starting at PNW 5-6	~0.073 mg/kg/d (0.01 mg/d)	NC					
					~53.2 mg/kg/d (7.1 mg/d)	NC					
	Odum et al. 1999b	Alderley Park rat	SC	11 days starting at PNW 5-6	~0.052 mg/kg/d (0.01 mg/d)	NC				NC	
					~37.4 mg/kg/d (7.1 mg/d)	NC				NC	
			Gavage		100 mg/kg/d	Increased lobules I, reduced TEBs				NC	
PCBs (Arachlor 1221/1254)	Brown and Lamartiniere 1995	Sprague Dawley rat	Gavage	PND 25, 27, 29, 31	0.025 mg/kg/d	NC			NC	NC	
PFOA	Yang et al. 2009	BALB/c mouse	Gavage	4 weeks starting at PND 21	0.1 mg/kg/d	NC					
					1 mg/kg/d	NC	Delayed			Reduced	
					5 mg/kg/d	Inhibited growth/differentiation and proliferation	No opening by 7 weeks			Reduced	
					10 mg/kg/d	Inhibited growth/differentiation; proliferation not evaluated	No opening			Reduced	
	Yang et al. 2009	C57BL/6 mouse	Gavage	4 weeks starting at PND 21	1 mg/kg/d	NC	NC			Increased	
					5 mg/kg/d	Increased differentiation and proliferation	No opening			NC	
					10 mg/kg/d	Inhibited growth/ differentiation	No opening			Reduced	

[a] unless noted, all mammary gland evaluations in studies with pubertal dosing were conducted within 24 hours of the end of the dosing period.

Supplemental Material Table 1: Studies describing altered mammary gland development: c) males-in utero/perinatal exposure

Compound	Study	Animal	ROE	Dosing interval	Doses	MG effects	Pubertal timing	AGD	Testes wt	Prostate wt	Sperm dev
**Endogenous or pharmaceutical hormones**											
Ethinyl estradiol	NTP 2010 (see also Latendresse et al 2009)	Sprague Dawley rat	Diet	GD 0-PND 21 or 50	2 ppb	NC				NC	
					10 ppb	NC				NC	
					50 ppb	Ductal hyperplasia at PND 50				Reduced (ventral)	
MER-25 (ethamoxytriphetol), an antiestrogen	Jean-Faucher et al. 1977	Swiss mouse	SC	GD 13-18	5 mg/d	NC at GD 19					
**Dietary components**											
Genistien	You et al. 2002	Sprague Dawley rat	Diet	GD1 - PND 22	300 ppm	NC					
					800 ppm	Increased branching, increased epithelial proliferation at PND 22					
	Delclos et al. 2001	Sprague Dawley rat	Diet	GD7 - PND50	5 ppm	NC	NC	NC	NC	NC	NC
					25 ppm	Hypertrophy at PND 50	NC	NC	NC	NC	NC
					100 ppm	" "	NC	NC	NC	NC	NC
					250 ppm	Hypertrophy and hyperplasia at PND 50	NC	NC	NC	NC	NC
					625 ppm	" "	NC	NC	NC	NC	NC
					1250 ppm	" "	NC	NC	NC	Reduced (ventral)	Abnormal spermatogenesis
**Environmental chemicals**											
Antiandrogen mixture[a]	Rider et al. 2008	SD rats	Gavage	GD 14-18	25%[a]	NC		NC	NC	NC	
					50%[a]	Retention of areolae at PND 13		NC	NC	NC	
					75%[a]	" "		Reduced	NC	NC	
					100%[a]	" "		Reduced	NC	Reduced (ventral)	
Dibutyl phthalate	Lee et al. 2004	CD(SD)IGS rat	Diet	GD 15 - PND 21	20 ppm	NC	NC	NC	NC		Reduced spermatocyte
					200 ppm	NC	NC	NC	NC		
					2000 ppm	NC	NC	NC	NC		
					10000 ppm	Retention of nipples/areolae at PND 14	NC	Reduced	Reduced		
Methoxychlor	You et al. 2002	Sprague Dawley rat	Diet	GD1 - PND 22	800 ppm	Increased area, branches, TEBs, LBs, increased cell division in epithelium at PND 22					

[a] Rider et al. 2008 investigated the effects of a mixture of 7 antiandrogens. The highest dose comprised 150 mg/kg/d each BBP, DBP, and DEHP, 35 mg/kg/d prochloraz, 20 mg/kg/d linuron, and 150 mg/kg/d each vinclozolin and procymidone. Doses are reported as percentages of this highest dose.

Supplemental Material, Table 2. Female mammary gland outcomes after developmental environmental exposures: rodent-human concordance for selected agents (fully cited version of Table 1)

Environmental Factor	Human Study MG Outcomes			Animal Study MG Outcomes		
	**Development**	**Lactation**	**Cancer Risk**	**Development**	**Lactation**	**Cancer Susceptibility**
Hormonal Milieu: dosing (animals) or surrogates (humans)	• Earlier breast development associated with higher birth weight (Olivo-Marston et al. 2010)<br>• Earlier breast development associated with higher BMI (Lee et al. 2007; Rosenfield et al. 2009; reviewed in Kaplowitz 2008)	•Mid-pregnancy androgen levels inversely related to breastfeeding at 3 and 6 months (Carlsen et al. 2010)	• Increased BC risk with high birth weight or length, advanced maternal or paternal age, decreased risk with pre-eclampsia, eclampsia, twinning (reviewed in Xue and Michels 2007)<br>• Childhood BMI inversely correlated with BC risk (reviewed in Ruder et al. 2008)	• Range of effects reported (see Supplemental Material, Table 1) (Cabanes et al. 2004; Hilakivi-Clarke et al. 1998; Hilakivi-Clarke et al. 1997; Latendresse et al. 2009, and others)	EE2:<br>• Impaired lactation (assessed via pre-weaning pup weights) with continuous exposure; no effect if dosed only in utero and through weaning (NTP 2010)	• Neonatal estrogen or androgen exposure increased MG tumors in MMTV+ mice (Lopez et al. 1988; Mori et al. 1976; Mori et al. 1979)
DES			• Mixed evidence: Palmer et al. (2006) found increased BC risk after age 40 from in utero exposure, while Verloop et al. (2010) found no such association.	• Range of effects reported (see Supplemental Material, Table 1) (Fielden et al. 2002; Hovey et al. 2005; Tomooka and Bern 1982, and others)	• Impaired lactation in rats exposed while pregnant (Browning et al. 1958)	• Gestational exposure increased spontaneous MG tumors in rats (Rothschild et al. 1987) and susceptibility to carcinogen challenge (Boylan and Calhoon 1979)<br>•Lower doses increased susceptibility to carcinogen challenge (Ninomiya et al. 2007); high dose decreased MG tumors (Lamartiniere and Holland 1992; Ninomiya et al. 2007)
Genistein/soy	• Lower urinary phytoestrogen biomarker concentrations associated with earlier breast development (Wolff et al. 2008)		• Mixed evidence, some suggesting reduced risk, especially from childhood or adolescent intake (Korde et al. 2009; Lee et al. 2009; Nagata 2010; Trock et al. 2006; Wu et al. 2008, and others)	• Range of effects reported (see Supplemental Material, Table 1) (Delclos et al. 2001; Hilakivi-Clarke et al. 2002; Padilla-Banks et al. 2006, and others)	• Impaired lactation (assessed via pre-weaning pup weights) in offspring dosed in utero through weaning or continuously (NTP 2008)	• Exposure increased or decreased susceptibility to carcinogen challenge, risk of spontaneous tumors, depending on timing (reviewed in Warri et al. 2008)

Supplemental Material, Table 2. Female mammary gland outcomes after developmental environmental exposures: rodent-human concordance for selected agents (fully cited version of Table 1) (continued)

Environmental Factor	Human Study MG Outcomes			Animal Study MG Outcomes		
	**Development**	**Lactation**	**Cancer Risk**	**Development**	**Lactation**	**Cancer Susceptibility**
DDT/DDE		• Mixed results re: association between shortened lactation, high serum DDT/ DDE levels (Cupul-Uicab et al. 2008; Gladen and Rogan 1995; Rogan et al. 1987)	• Strong association with blood DDT levels at first birth in women who were adolescents during peak DDT use years (Cohn et al. 2007)<br>•Little evidence of increased risk seen in studies limited to adult exposures (reviewed in Brody et al. 2007)	• Range of effects reported (see Supplemental Material, Table 1) (Brown and Lamartiniere 1995)	No lactation effect observed in rats dosed 5 weeks pre-weaning through lactation (Kornbrust et al. 1986)	
Dioxins/furans (TCDD/PCDD/ PCDF)	• High level of dioxin-like activity in serum associated with lower likelihood of completed breast development (Den Hond et al. 2002)<br>• Higher prenatal and/or lactational PCDD/F exposure associated with delayed initiation of self-reported breast development (Leijs et al. 2008)		• Some analyses show increased BC risk with higher dioxin exposure after an industrial accident (Pesatori et al. 2009; Warner et al. 2002)<br>• Limited evidence of increased risk from occupational, other exposures (reviewed in Brody et al. 2007)	• Range of effects reported (see Supplemental Material, Table 1) (Brown et al. 1998; Fenton et al. 2002; Lewis et al. 2001)	• Impaired lactation in dams exposed while pregnant (Lew et al. 2009; Vorderstrasse et al. 2004)	• Prenatal exposure increases susceptibility to carcinogen challenge in rats (Brown et al. 1998; Desaulniers et al. 2001)

Note: This table provides some examples of concordance between rodent and human studies for mammary gland effects. In some cases, findings are mixed or conflicting. In human studies, exposure measures are often imprecise.
BC, breast cancer; BMI, body mass index; DDE, dichlorodiphenyltrichloroethylene; DDT, dichlorodiphenyltrichloroethane; DES, diethylstilbestrol; DMBA, dimethylbenzanethrene; EE2,ethinylestradiol; MG, mammary gland; MMTV+, mouse mammary tumor virus-positive; PCDD, polychlorinated dibenzo-p-dioxin; PCDF, polychlorinated dibenzofuran; TCDD, 2,3,7,8-tetrachlorodibenzo-p-dioxin

Supplemental Material References:

Boylan ES, Calhoon RE. 1979. Mammary tumorigenesis in the rat following prenatal exposure to diethylstilbestrol and postnatal treatment with 7,12-dimethylbenz[a]anthracene. J Toxicol Environ Health 5(6): 1059-1071.

Brody JG, Moysich KB, Humblet O, Attfield KR, Beehler GP, Rudel RA. 2007. Environmental pollutants and breast cancer: epidemiologic studies. Cancer 109(12 Suppl): 2667-2711.

Brown NM, Lamartiniere CA. 1995. Xenoestrogens alter mammary gland differentiation and cell proliferation in the rat. Environ Health Perspect 103(7-8): 708-713.

Brown NM, Manzolillo PA, Zhang JX, Wang J, Lamartiniere CA. 1998. Prenatal TCDD and predisposition to mammary cancer in the rat. Carcinogenesis 19(9): 1623-1629.

Browning CB, Parrish DB, Fountaine FC. 1958. Effect of feeding low levels of diethylstilbestrol on gestation and lactation of rats. J Nutr 66(3): 321-332.

Cabanes A, Wang M, Olivo S, DeAssis S, Gustafsson JA, Khan G, et al. 2004. Prepubertal estradiol and genistein exposures up-regulate BRCA1 mRNA and reduce mammary tumorigenesis. Carcinogenesis 25(5): 741-748.

Carlsen SM, Jacobsen G, Vanky E. 2010. Mid-pregnancy androgen levels are negatively associated with breastfeeding. Acta Obstet Gynecol Scand 89(1): 87-94.

Cohn BA, Wolff MS, Cirillo PM, Sholtz RI. 2007. DDT and Breast Cancer in Young Women: New Data on the Significance of Age at Exposure. Environ Health Perspect 115(10): 1406-1414.

Colerangle JB, Roy D. 1996. Exposure of environmental estrogenic compound nonylphenol to noble rats alters cell kinetics in the mammary gland. Endocrine 4: 115-122.

Colerangle JB, Roy D. 1997. Profound effects of the weak environmental estrogen-like chemical bisphenol A on the growth of the mammary gland of Noble rats. The Journal of Steroid Biochemistry and Molecular Biology 60(1-2): 153-160.

Cotroneo MS, Wang J, Fritz WA, Eltoum IE, Lamartiniere CA. 2002. Genistein action in the prepubertal mammary gland in a chemoprevention model. Carcinogenesis 23(9): 1467-1474.

Cupul-Uicab LA, Gladen BC, Hernandez-Avila M, Weber JP, Longnecker MP. 2008. DDE, a degradation product of DDT, and duration of lactation in a highly exposed area of Mexico. Environ Health Perspect 116(2): 179-183.

Delclos KB, Bucci TJ, Lomax LG, Latendresse JR, Warbritton A, Weis CC, et al. 2001. Effects of dietary genistein exposure during development on male and female CD (Sprague-Dawley) rats. Reprod Toxicol 15(6): 647-663.

Den Hond E, Roels HA, Hoppenbrouwers K, Nawrot T, Thijs L, Vandermeulen C, et al. 2002. Sexual maturation in relation to polychlorinated aromatic hydrocarbons: Sharpe and Skakkebaek's hypothesis revisited. Environ Health Perspect 110(8): 771-776.

Desaulniers D, Leingartner K, Russo J, Perkins G, Chittim BG, Archer MC, et al. 2001. Modulatory effects of neonatal exposure to TCDD, or a mixture of PCBs, p,p'-DDT, and p-p'-DDE, on methylnitrosourea-induced mammary tumor development in the rat. Environ Health Perspect 109(7): 739-747.

Doherty L, Bromer J, Zhou Y, Aldad T, Taylor H. 2010. In utero exposure to diesthylstilbesterol (DES) or bisphenol-A (BPA) increases EZH2 expression in the mammary gland: An epigenetic mechanism linking endocrine disruptors to breast cancer. Horm Canc; doi: 10.1007/s12672-010-0015-9 [Online 15 May 2010].

Durando M, Kass L, Piva J, Sonnenschein C, Soto AM, Luque EH, et al. 2007. Prenatal bisphenol A exposure induces preneoplastic lesions in the mammary gland in Wistar rats. Environ Health Perspect 115(1): 80-86.

Enoch RR, Stanko JP, Greiner SN, Youngblood GL, Rayner JL, Fenton SE. 2007. Mammary gland development as a sensitive end point after acute prenatal exposure to an atrazine metabolite mixture in female Long-Evans rats. Environ Health Perspect 115(4): 541-547.

Fenton SE, Hamm JT, Birnbaum LS, Youngblood GL. 2002. Persistent abnormalities in the rat mammary gland following gestational and lactational exposure to 2,3,7,8-tetrachlorodibenzo-p-dioxin (TCDD). Toxicol Sci 67(1): 63-74.

Fielden MR, Fong CJ, Haslam SZ, Zacharewski TR. 2002. Normal mammary gland morphology in pubertal female mice following in utero and lactational exposure to genistein at levels comparable to human dietary exposure. Toxicol Lett 133(2-3): 181-191.

Foster WG, Mirshokraei P, Holloway AC, Zhang B. 2008. Developmental and lactational exposure to environmentally relevant concentrations of dieldrin does not alter pregnancy outcome and mammary gland morphology in BALB/c mice. Environ Res 108(1): 21-27.

Fritz WA, Coward L, Wang J, Lamartiniere CA. 1998. Dietary genistein: perinatal mammary cancer prevention, bioavailability and toxicity testing in the rat. Carcinogenesis 19(12): 2151-2158.

Gladen BC, Rogan WJ. 1995. DDE and shortened duration of lactation in a northern Mexican town. Am J Public Health 85(4): 504-508.

Hilakivi-Clarke L, Cho E, Cabanes A, DeAssis S, Olivo S, Helferich W, et al. 2002. Dietary modulation of pregnancy estrogen levels and breast cancer risk among female rat offspring. Clin Cancer Res 8(11): 3601-3610.

Hilakivi-Clarke L, Cho E, Clarke R. 1998. Maternal genistein exposure mimics the effects of estrogen on mammary gland development in female mouse offspring. Oncol Rep 5(3): 609-616.

Hilakivi-Clarke L, Cho E, Raygada M, Kenney N. 1997a. Alterations in mammary gland development following neonatal exposure to estradiol, transforming growth factor alpha, and estrogen receptor antagonist ICI 182,780. J Cell Physiol 170(3): 279-289.

Hilakivi-Clarke L, Clarke R, Onojafe I, Raygada M, Cho E, Lippman M. 1997b. A maternal diet high in n - 6 polyunsaturated fats alters mammary gland development, puberty onset, and breast cancer risk among female rat offspring. Proc Natl Acad Sci U S A 94(17): 9372-9377.

Hovey RC, Asai-Sato M, Warri A, Terry-Koroma B, Colyn N, Ginsburg E, et al. 2005. Effects of neonatal exposure to diethylstilbestrol, tamoxifen, and toremifene on the BALB/c mouse mammary gland. Biol Reprod 72(2): 423-435.

Ip C, Banni S, Angioni E, Carta G, McGinley J, Thompson HJ, et al. 1999. Conjugated linoleic acid-enriched butter fat alters mammary gland morphogenesis and reduces cancer risk in rats. J Nutr 129(12): 2135-2142.

Jean-Faucher C, Berger M, de Turckheim M, Veyssiere G, Jean C. 1977. Effects of an antioestrogen (MER-25) on sexual and mammary gland morphogenesis of the mouse fetus. J Reprod Fertil 51(2): 481-482.

Jenkins S, Raghuraman N, Eltoum I, Carpenter M, Russo J, Lamartiniere CA. 2009. Oral exposure to bisphenol a increases dimethylbenzanthracene-induced mammary cancer in rats. Environ Health Perspect 117(6): 910-915.

Kaplowitz PB. 2008. Link between body fat and the timing of puberty. Pediatrics 121 Suppl 3: S208-217.

Kodavanti PRS, Coburn CG, Moser VC, MacPhail RC, Fenton SE, Stoker TE, et al. 2010. Developmental exposure to a commercial PBDE mixture, DE-71: neurobehavioral, hormonal, and reproductive effects. Toxicol Sci.

Korde LA, Wu AH, Fears T, Nomura AM, West DW, Kolonel LN, et al. 2009. Childhood soy intake and breast cancer risk in Asian American women. Cancer Epidemiol Biomarkers Prev 18(4): 1050-1059.

Kornbrust D, Gillis B, Collins B, Goehl T, Gupta B, Schwetz B. 1986. Effects of 1,1-dichloro-2,2-bis[p-chlorophenyl]ethylene (DDE) on lactation in rats. J Toxicol Environ Health 17(1): 23-36.

Lamartiniere CA, Holland MB. 1992. Neonatal diethylstilbestrol prevents spontaneously developing mammary tumors. In: Proceedings of the 1st International Symposium on Hormonal Carcinogenesis, (Li JJ, Nandi S, Li SA, eds). New York:Springer-Verlag, 305-308.

Latendresse JR, Bucci TJ, Olson G, Mellick P, Weis CC, Thorn B, et al. 2009. Genistein and ethinyl estradiol dietary exposure in multigenerational and chronic studies induce similar proliferative lesions in mammary gland of male Sprague-Dawley rats. Reprod Toxicol 28(3): 342-353.

Lee JM, Appugliese D, Kaciroti N, Corwyn RF, Bradley RH, Lumeng JC. 2007. Weight status in young girls and the onset of puberty. Pediatrics 119(3): 624-630.

Lee KY, Shibutani M, Takagi H, Kato N, Takigami S, Uneyama C, et al. 2004. Diverse developmental toxicity of di-n-butyl phthalate in both sexes of rat offspring after maternal exposure during the period from late gestation through lactation. Toxicology 203(1-3): 221-238.

Lee SA, Shu XO, Li H, Yang G, Cai H, Wen W, et al. 2009. Adolescent and adult soy food intake and breast cancer risk: results from the Shanghai Women's Health Study. Am J Clin Nutr 89(6): 1920-1926.

Leijs MM, Koppe JG, Olie K, van Aalderen WM, Voogt P, Vulsma T, et al. 2008. Delayed initiation of breast development in girls with higher prenatal dioxin exposure; a longitudinal cohort study. Chemosphere 73(6): 999-1004.

Lew BJ, Collins LL, O'Reilly MA, Lawrence BP. 2009. Activation of the aryl hydrocarbon receptor during different critical windows in pregnancy alters mammary epithelial cell proliferation and differentiation. Toxicol Sci 111(1): 151-162.

Lewis BC, Hudgins S, Lewis A, Schorr K, Sommer R, Peterson RE, et al. 2001. In utero and lactational treatment with 2,3,7,8-tetrachlorodibenzo-p-dioxin impairs mammary gland differentiation but does not block the response to exogenous estrogen in the postpubertal female rat. Toxicol Sci 62(1): 46-53.

Lopez J, Ogren L, Verjan R, Talamantes F. 1988. Effects of perinatal exposure to a synthetic estrogen and progestin on mammary tumorigenesis in mice. Teratology 38(2): 129-134.

Markey CM, Luque EH, Munoz de Toro M, Sonnenschein C, Soto AM. 2001. In utero exposure to bisphenol A alters the development and tissue organization of the mouse mammary gland. Biol Reprod 65(4): 1215-1215.

Moon HJ, Han SY, Shin JH, Kang IH, Kim TS, Hong JH, et al. 2007. Gestational exposure to nonylphenol causes precocious mammary gland development in female rat offspring. J Reprod Dev 53(2): 333-344.

Moral R, Wang R, Russo IH, Lamartiniere CA, Pereira J, Russo J. 2008. Effect of prenatal exposure to the endocrine disruptor bisphenol A on mammary gland morphology and gene expression signature. J Endocrinol 196(1): 101-112.
Moral R, Wang R, Russo IH, Mailo DA, Lamartiniere CA, Russo J. 2007. The plasticizer butyl benzyl phthalate induces genomic changes in rat mammary gland after neonatal/prepubertal exposure. BMC Genomics 8(1): 453-453.
Mori T, Bern HA, Mills KT, Young PN. 1976. Long-term effects of neonatal steroid exposure on mammary gland development and tumorigenesis in mice. J Natl Cancer Inst 57(5): 1057-1062.
Mori T, Nagasawa H, Bern HA. 1979. Long-term effects of perinatal exposure to hormones on normal and neoplastic mammary growth in rodents: a review. J Environ Pathol Toxicol 3(1-2): 191-205.
Munoz-de-Toro M, Markey CM, Wadia PR, Luque EH, Rubin BS, Sonnenschein C, et al. 2005. Perinatal exposure to bisphenol-A alters peripubertal mammary gland development in mice. Endocrinology 146(9): 4138-4147.
Murray TJ, Maffini MV, Ucci AA, Sonnenschein C, Soto AM. 2007. Induction of mammary gland ductal hyperplasias and carcinoma in situ following fetal bisphenol A exposure. Reprod Toxicol 23(3): 383-390.
Murrill WB, Brown NM, Zhang JX, Manzolillo PA, Barnes S, Lamartiniere CA. 1996. Prepubertal genistein exposure suppresses mammary cancer and enhances gland differentiation in rats. Carcinogenesis 17(7): 1451-1457.
Nagata C. 2010. Factors to consider in the association between soy isoflavone intake and breast cancer risk. J Epidemiol 20(2): 83-89.
Nikaido Y, Yoshizawa K, Danbara N, Tsujita-Kyutoku M, Yuri T, Uehara N, et al. 2004. Effects of maternal xenoestrogen exposure on development of the reproductive tract and mammary gland in female CD-1 mouse offspring. Reprod Toxicol 18(6): 803-811.
Ninomiya K, Kawaguchi H, Souda M, Taguchi S, Funato M, Umekita Y, et al. 2007. Effects of neonatally administered diethylstilbestrol on induction of mammary carcinomas induced by 7, 12-dimethylbenz(a)anthracene in female rats. Toxicol Pathol 35(6): 813-818.
NTP. 2008. NTP Technical Report on the Multigenerational Reproductive Toxicology Study of Genistein in Sprague-Dawley Rats. NTP TR 539. Research Triangle Park, NC:National Toxicology Program.
NTP. 2010. NTP Technical Report on the Multigenerational Reproductive Toxicology Study of Ethinyl Estradiol in Sprague-Dawley Rats NTP TR 547; NIH 10-5888. Research Triangle Park, NC:National Toxicology Program.
Odum J, Pyrah IT, Foster JR, Van Miller JP, Joiner RL, Ashby J. 1999a. Comparative activities of p-nonylphenol and diethylstilbestrol in noble rat mammary gland and uterotrophic assays. Regul Toxicol Pharmacol 29(2 Pt 1): 184-195.
Odum J, Pyrah IT, Soames AR, Foster JR, Van Miller JP, Joiner RL, et al. 1999b. Effects of p-nonylphenol (NP) and diethylstilboestrol (DES) on the Alderley Park (Alpk) rat: comparison of mammary gland and uterus sensitivity following oral gavage or implanted mini-pumps. J Appl Toxicol 19(5): 367-378.
Olivo-Marston S, Graubard BI, Visvanathan K, Forman MR. 2010. Gender-specific differences in birthweight and the odds of puberty: NHANES III, 1988-94. Paediatr Perinat Epidemiol 24(3): 222-231.

Padilla-Banks E, Jefferson WN, Newbold RR. 2006. Neonatal exposure to the phytoestrogen genistein alters mammary gland growth and developmental programming of hormone receptor levels. Endocrinology 147(10): 4871-4882.

Palmer JR, Wise LA, Hatch EE, Troisi R, Titus-Ernstoff L, Strohsnitter W, et al. 2006. Prenatal diethylstilbestrol exposure and risk of breast cancer. Cancer Epidemiol Biomarkers Prev 15(8): 1509-1514.

Pesatori AC, Consonni D, Rubagotti M, Grillo P, Bertazzi PA. 2009. Cancer incidence in the population exposed to dioxin after the "Seveso accident": twenty years of follow-up. Environ Health 8: 39.

Rayner JL, Enoch RR, Fenton SE. 2005. Adverse effects of prenatal exposure to atrazine during a critical period of mammary gland growth. Toxicol Sci 87(1): 255-266.

Rider CV, Furr J, Wilson VS, Gray LE. 2008. A mixture of seven antiandrogens induces reproductive malformations in rats. Int J Androl 31(2): 249-262.

Rogan WJ, Gladen BC, McKinney JD, Carreras N, Hardy P, Thullen J, et al. 1987. Polychlorinated biphenyls (PCBs) and dichlorodiphenyl dichloroethene (DDE) in human milk: effects on growth, morbidity, and duration of lactation. Am J Public Health 77(10): 1294-1297.

Rosenfield RL, Lipton RB, Drum ML. 2009. Thelarche, pubarche, and menarche attainment in children with normal and elevated body mass index. Pediatrics 123(1): 84-88.

Rothschild TC, Boylan ES, Calhoon RE, Vonderhaar BK. 1987. Transplacental effects of diethylstilbestrol on mammary development and tumorigenesis in female ACI rats. Cancer Res 47(16): 4508-4516.

Ruder EH, Dorgan JF, Kranz S, Kris-Etherton PM, Hartman TJ. 2008. Examining breast cancer growth and lifestyle risk factors: early life, childhood, and adolescence. Clin Breast Cancer 8(4): 334-342.

Santell RC, Chang YC, Nair MG, Helferich WG. 1997. Dietary genistein exerts estrogenic effects upon the uterus, mammary gland and the hypothalamic/pituitary axis in rats. J Nutr 127(2): 263-269.

Tan KP, Chen J, Ward WE, Thompson LU. 2004. Mammary gland morphogenesis is enhanced by exposure to flaxseed or its major lignan during suckling in rats. Exp Biol Med (Maywood) 229(2): 147-157.

Tomooka Y, Bern HA. 1982. Growth of mouse mammary glands after neonatal sex hormone treatment. J Natl Cancer Inst 69(6): 1347-1352.

Tou JC, Thompson LU. 1999. Exposure to flaxseed or its lignan component during different developmental stages influences rat mammary gland structures. Carcinogenesis 20(9): 1831-1835.

Trock BJ, Hilakivi-Clarke L, Clarke R. 2006. Meta-analysis of soy intake and breast cancer risk. J Natl Cancer Inst 98(7): 459-471.

Vandenberg LN, Maffini MV, Wadia PR, Sonnenschein C, Rubin BS, Soto AM. 2007. Exposure to environmentally relevant doses of the xenoestrogen bisphenol-A alters development of the fetal mouse mammary gland. Endocrinology 148(1): 116-127.

Verloop J, van Leeuwen FE, Helmerhorst TJ, van Boven HH, Rookus MA. 2010. Cancer risk in DES daughters. Cancer Causes Control 21(7): 999-1007.

Vorderstrasse BA, Fenton SE, Bohn AA, Cundiff JA, Lawrence BP. 2004. A novel effect of dioxin: exposure during pregnancy severely impairs mammary gland differentiation. Toxicol Sci 78(2): 248-257.

Ward WE, Jiang FO, Thompson LU. 2000. Exposure to flaxseed or purified lignan during lactation influences rat mammary gland structures. Nutr Cancer 37(2): 187-192.

Warner M, Eskenazi B, Mocarelli P, Gerthoux PM, Samuels S, Needham L, et al. 2002. Serum dioxin concentrations and breast cancer risk in the Seveso Women's Health Study. Environ Health Perspect 110(7): 625-628.

Warner MR. 1976. Effect of various doses of estrogen to BALB/cCrgl neonatal female mice on mammary growth and branching at 5 weeks of age. Cell Tissue Kinet 9(5): 429-438.

Warri A, Saarinen NM, Makela S, Hilakivi-Clarke L. 2008. The role of early life genistein exposures in modifying breast cancer risk. Br J Cancer 98(9): 1485-1493.

White SS, Calafat AM, Kuklenyik Z, Villanueva L, Zehr RD, Helfant L, et al. 2007. Gestational PFOA exposure of mice is associated with altered mammary gland development in dams and female offspring. Toxicol Sci 96(1): 133-144.

White SS, Kato K, Jia LT, Basden BJ, Calafat AM, Hines EP, et al. 2009. Effects of perfluorooctanoic acid on mouse mammary gland development and differentiation resulting from cross-foster and restricted gestational exposures. Reprod Toxicol 27(3-4): 289-298.

Wolff MS, Britton JA, Boguski L, Hochman S, Maloney N, Serra N, et al. 2008. Environmental exposures and puberty in inner-city girls. Environ Res 107(3): 393-400.

Wu AH, Koh WP, Wang R, Lee HP, Yu MC. 2008. Soy intake and breast cancer risk in Singapore Chinese Health Study. Br J Cancer 99(1): 196-200.

Xue F, Michels KB. 2007. Intrauterine factors and risk of breast cancer: a systematic review and meta-analysis of current evidence. The Lancet Oncology 8(12): 1088-1100.

Yang C, Tan YS, Harkema JR, Haslam SZ. 2009. Differential effects of peripubertal exposure to perfluorooctanoic acid on mammary gland development in C57Bl/6 and Balb/c mouse strains. Reprod Toxicol 27(3-4): 299-306.

Yin H, Ito A, Bhattacharjee D, Hoshi M. 2006. A comparative study on the protective effects of 17beta-estradiol, biochanin A and bisphenol A on mammary gland differentiation and tumorigenesis in rats. Indian J Exp Biol 44(7): 540-546.

You L, Casanova M, Bartolucci EJ, Fryczynski MW, Dorman DC, Everitt JI, et al. 2002. Combined effects of dietary phytoestrogen and synthetic endocrine-active compound on reproductive development in Sprague-Dawley rats: genistein and methoxychlor. Toxicol Sci 66(1): 91-104.
